# Thermoresponsiveness Across the Physiologically Accessible Range: Effect of Surfactant, Cross-Linker, and Initiator Content on Size, Structure, and Transition Temperature of Poly(*N*-isopropylmethacrylamide) Microgels

**DOI:** 10.1021/acsomega.4c02115

**Published:** 2024-08-12

**Authors:** Danielle Winning, Jacek K. Wychowaniec, Bing Wu, Andreas Heise, Brian J. Rodriguez, Dermot F. Brougham

**Affiliations:** † School of Chemistry, University College Dublin, Belfield, Dublin 4, Ireland; ‡ AO Research Institute Davos, Clavadelerstrasse 8, 7270 Davos, Switzerland; § Dutch-Belgian Beamline (DUBBLE), European Synchrotron Radiation Facility (ESRF), 71 Avenue Des Martyrs, CS 40220, Grenoble 38043, France; ∥ Department of Chemistry, Royal College of Surgeons in Ireland, Dublin 9, Ireland; ⊥ Conway Institute of Biomolecular and Biomedical Research, University College Dublin, Belfield, Dublin 4, Ireland; # School of Physics, University College Dublin, Belfield, Dublin 4, Ireland

## Abstract

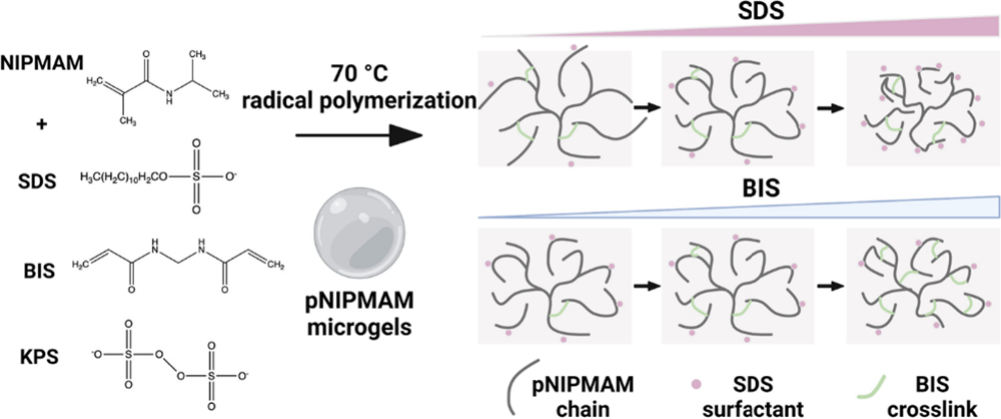

The influence of surfactant, cross-linker, and initiator on the final structure and thermoresponse of poly(*N*-isopropylmethacrylamide) (pNIPMAM) microgels was evaluated. The goals were to control particle size (into the nanorange) and transition temperature (across the physiologically accessible range). The concentration of the reactants used in the synthesis was varied, except for the monomer, which was kept constant. The thermoresponsive suspensions formed were characterized by dynamic light scattering, small-angle X-ray scattering, atomic force microscopy, and rheology. Increasing surfactant, sodium dodecyl sulfate content, produced smaller microgels, as expected, into the nanorange and with greater internal entanglement, but with no change in phase transition temperature (LCST), which is contrary to previous reports. Increasing cross-linker, *N*,*N*-methylenebis acrylamide, content had no impact on particle size but reduced particle deformability and, again contrary to previous reports of decreases, progressively increased the LCST from 39 to 46 °C. The unusual LCST trends were confirmed using different rheological techniques. Initiator, potassium persulfate, content was found to weakly influence the outcomes. An optimized content was identified that provides functional nanogels in the 100 nm (swollen) size range with controlled LCST, just above physiological temperature. The study contributes chemistry-derived design rules for thermally responsive colloidal particles with physiologically accessible LCST for a variety of biomedical and soft robotics applications.

## Introduction

**Figure 1:**
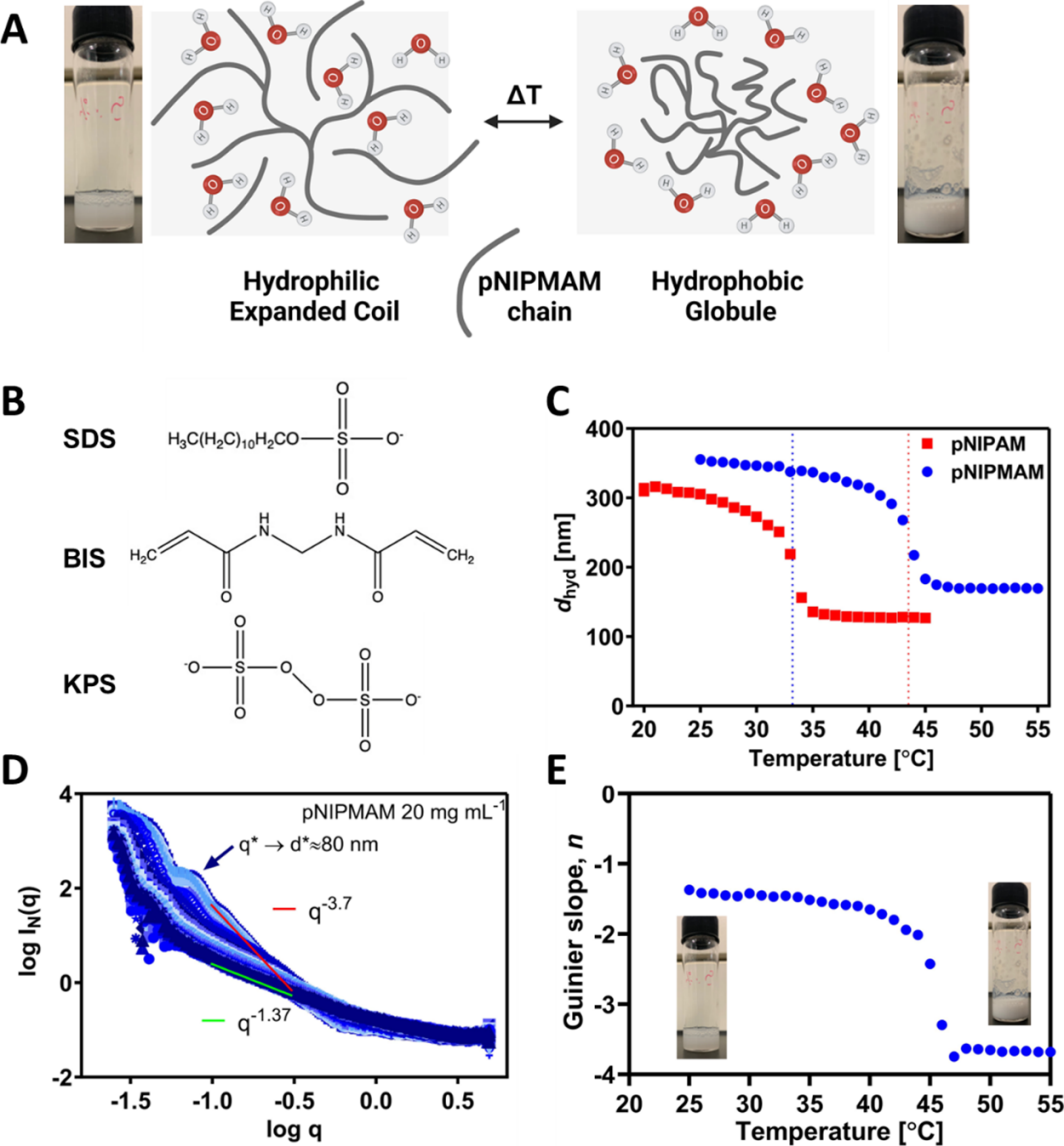
(A) Schematic swollen (left) and collapsed (right) structures of LCST-type thermoresponsive polymer microgels showing a temperature reversible coil-to-globule transition. (B) Chemical structures of SDS, BIS, and KPS. (C) Hydrodynamic size, *d*_hyd_, as a function of the temperature for pNIPAM (red) and pNIPMAM (blue) microgels. Samples were prepared using 1.3 mM SDS, 1.4 mol % BIS, and 3.4 wt % KPS. Samples were measured by DLS at polymer concentration of 5 mg mL^–1^ and at 1 °C increments across the temperature range, with 300 s equilibration time at each temperature. (D) SAXS measured for the same pNIPMAM microgels at 20 mg mL^–1^ between 25 and 55 °C, at 1 °C increments with 300 s equilibration time. Intensity is depicted as a gradient in color in the plot. Log *I*(*q*) as a function of log *q* data was fitted with lines to extract Guinier’s slope (*n*), which are represented as a function of temperature in (E). Photograph insets in (A,E) show pNIPMAM microgel suspensions at 25 (left) and 50 °C (right) at 10 mg mL^–1^ at which concentration, the transition is more apparent visually.

Thermoresponsive polymers are used as components of biomaterials to generate temperature-induced changes in miscibility^1^ that can, in principle, provide burst-free drug- or cell-release and even robotic motion.^2^ These possibilities usually arise due to lower critical solution temperature (LCST) phase transitions.^3,4^ For acrylamide-based polymers, this coil-to-globule transition occurs through breakage of hydrogen bonds between N–H and C=O groups of the polymer chains and the surrounding water molecules, which are then expelled.^5,6^ Consequently, polymer–polymer interactions dominate, leading to a collapse of the chain conformation and formation of insoluble hydrophobic globules (Figure 1A).^7−9^

Poly(*N*-isopropylacrylamide) (pNIPAM), with a phase transition *c.* 32 °C,^6,10^ is a very commonly used LCST material as it is responsive above room temperature, is biocompatible, has excellent mechanical properties, and is relatively easy and inexpensive to synthesize.^11^ Microgels formed from pNIPAM have been used in a range of biomedical applications, for example as porous films for cell culture which enhance cell proliferation through improved distribution of nutrients and oxygen at *T* > *LCST*(4,12) and for controlled detachment of adsorbed cells through temperature cycling.^4,12−14^ pNIPAM copolymers have also been exploited for localized delivery of chemotherapeutics and proteins from the micellar core on chain collapse at the transition.^15^ On the basis of SAXS, SANS, and DLS measurements, the consensus is that microgels can loosely be considered as “core–shell” structures with a highly cross-linked inner part and progressively decreasing extent of cross-linking with distance from the center.^16−18^ However, as the transition temperature is below the physiological range, biomedical applications of pNIPAM are somewhat limited.

The LCST can be increased by increasing the hydrophilicity of the polymer chains either through copolymerization^19^ or by addition of hydrophilic chain groups or hydrophobic additives.^8^*N*-isopropylmethacrylamide (NIPMAM) is structurally similar to NIPAM, but with the addition of a methyl group at the polymerization site, it increases polarity and provides additional stabilization during polymerization. As a result, the LCST of pNIPMAM has been reported in the range of 38–44 °C.^9,20^ The synthesis is by free radical polymerization with the initiation and propagation of monomer units by charged initiator fragments to provide initial polymer chains, or nuclei, from which microgel particles subsequently form.^21^ Surfactants such as sodium dodecyl sulfate (SDS) have been used to provide size control and improve monodispersity. Difunctional monomers are often included to internally cross-link particles to improve cohesion and robustness.^22^

For pNIPAM, the swollen state hydrodynamic size, *d*_hyd_, has been shown to decrease with increasing SDS concentration in the reaction. Increasing the surfactant content is thought to increase the number of nuclei that form/persist. This is typically explained as due to surfactant molecules reducing surface tension that both promotes nucleation and reduces aggregation of nuclei.^23,24^ Decreasing *d*_hyd_ with increasing SDS concentration has also been reported for pNIPMAM microgels.^25,26^ Interestingly, using DLS data only, the authors of the first study^25^ showed a *c.* 2 °C increase in LCST to *c.* 45 °C when SDS was present, but no dependence of LCST on concentration. The effect was ascribed to less homogeneous cross-linking in the absence of surfactant.

Cross-linking typically increases the mechanical strength, reduces the loss of soluble polymer, and allows for efficient swelling/deswelling in the loading/release of encapsulated molecules. Hence it almost always utilized, with *N*,*N*-methylenebis acrylamide (BIS) the most common cross-linker. For growing pNIPMAM microgels, if only one of the two vinyl groups in BIS reacts, the molecule is simply incorporated as a monomer unit within a linear poly(NIPMAM) chain, while if both groups react, the molecule acts as a cross-linker/loop former in the growing network. Due to its increased solubility and higher reactivity, compared to the monofunctional monomers, BIS consumption in the early stages of the polymerization is rapid for both pNIPAM^27,28^ and pNIPMAM,^22^ resulting in the previously mentioned “core–shell” structures.^16−18^ Increased cross-linker content has been clearly shown to reduce the fraction of free “dangling” chains,^24,28,29^ i.e., to decrease the “shell” thickness.

For pNIPAM microgels, particle size is known to be relatively independent of cross-linker, usually BIS,^23,24^ content. While for pNIPMAM microgels, prepared in surfactant-free conditions, increased BIS content has been reported to reduce the extent of swelling, as expected, and to decrease the *LCST*, as measured using optical density only.^22,30^ Decreased LCST is perhaps unexpected given the strengthened H–bond interactions. For surfactant-containing pNIPMAM preparations, while there have been systematic studies into the effects of SDS,^25^ there is limited understanding of the effects of cross-linker or initiator content. These effects are difficult to predict, as the additional methyl group will change the hydrogen bonding but may also influence the kinetics of nucleation and growth that determine network structure.

In this study, the effects of surfactant (SDS), cross-linker (BIS), and initiator (potassium persulfate, KPS) content in the reaction on the colloidal, structural, and rheological properties of pNIPMAM were evaluated. It proved possible to prepare high-LCST microgels with size control into the nanorange and LCST tunable from just above physiological temperature to 46 °C. Care was taken to monitor structural changes by both DLS and SAXS, and trends in LCST were confirmed using different rheological techniques.

## Materials and Methods

### Schemes

All schemes and graphical abstracts were created with BioRender.com.

### Chemicals

*N*-isopropylacrylamide (NIPAM, 97%), *N*-isopropylmethacrylamide (NIPMAM, 97%), *N*,*N*-methylenebis acrylamide (BIS, 99%), sodium dodecyl sulfate (SDS, >98.5%), potassium persulfate (KPS, >99%).

### Polymer Microgel Synthesis

Microgels formed from pNIPAM/pNIPMAM were synthesized by free radical polymerization at 70 °C of NIPAM/NIPMAM (17.4 mmol) in water (100 mL) in the presence of *N*,*N*-methylenebis acrylamide (BIS), sodium dodecyl sulfate (SDS), and potassium persulfate (KPS) using an adapted synthesis by Berndt and Richtering.^31^ Initially, the monomer, surfactant, and cross-linker were heated under N_2_ flow with magnetic stirring in water (99 mL) at 70 °C for 1 h. Separately, the initiator was dissolved in 1 mL of water by thorough shaking. The polymerization was then initiated by addition of the initiator solution to the monomer solution. The reaction was allowed to proceed for 6 h under N_2_ flow at 70 °C. Resulting suspensions were dialyzed using 12–14 kDa MWCO dialysis tubing against 2 L Milli-Q water for 5 days with changing of water every 24 h. Extensive purification of the microgels was undertaken by dialysis. A time-dependent dialysis study for a pNIPMAM microgel preparation (1.3 mM SDS, 1.4 mol % BIS, and 3.4 wt % KPS) using DLS is shown in Figure S1. 1 mL portions of the suspensions were removed for each measurement and not replaced. The collapsed *d*_hyd_ values were found to rapidly settle to a low constant value. The swollen *d*_hyd_ was found to decrease with dialysis time up to 48 h, after which there was no further significant change. We suggest that all impurities affecting the processing/colloidal stabilization are removed by 5 days of dialysis. We further confirmed removal of significant low molecular weight material by the dialysis procedure using gravimetry. For a full-scale synthesis/prep after day 1 of dialysis, typically 10 mg of low mol weight material was removed. This was found to fall below 0.1 mg by day 5.

Samples were then freeze-dried and stored in enclosed containers at room temperature. Microgels formed from pNIPMAM were prepared under different synthetic conditions where the BIS content was varied from 0.0 to 11.2 mol %, SDS from 0.0 to 5.0 mM, and KPS from 0.0 to 6.0 wt % (Table 1). The average yield per synthesis was 91.4 ± 0.2% with respect to the initial monomer amount.

**Table 1:** Synthetic Conditions Used in Preparing the Three pNIPMAM Microgel Series

sample code	NIPMAM (mmol)	SDS (mM)^a^	BIS (mol %)^a^	KPS (wt %)^a^
SDS0	17.4	0.0	1.4	3.4
SDS1^b^		1.3		
SDS2		2.4		
SDS3		3.0		
SDS4		4.2		
BIS0	17.4	1.3	0.0	3.4
BIS1^b^			1.4	
BIS2			2.8	
BIS3			5.6	
BIS4			11.2	
KPS0	17.4	1.3	1.4	0.0
KPS1				0.5
KPS2				1.0
KPS3				2.0
KPS4^b^				3.4
KPS5				6.0
MG_OPT_^c^	17.4	5.0	1.8	3.4

a Amounts of SDS, BIS, and KPS are represented using the commonly ascribed units within literature.

b SDS1, BIS1, and KPS4 are the same suspension.

c MG_OPT_ is an optimized nanogel formulation based on results obtained from SDS, BIS, and KPS series.

### Temperature-dependent small-angle X-ray scattering (SAXS)

Synchrotron SAXS measurements presented in Figure S2 and Table 3 were measured at the I22 beamline in Diamond Light Source. The energy of the beam was 12.4 keV, corresponding to an X-ray wavelength of 0.1 nm. The samples were prepared as described above and injected into capillaries using a syringe. Samples were measured using a sample camera distance of ≈9 m, corresponding to an accessible momentum transfer vector range of 0.018 nm^–1^ < *q* = (4π/λ) sin(θ/2) < 1.7 nm^–1^, where θ is the scattering angle and λ is the wavelength of the incident photons. Calibration of the SAXS detector (Pilatus P3-2M, Dectris, Switzerland) was performed by using silver behenate powder. An empty capillary was used as the background and subtracted from all spectra, and data were reduced using the Dawn software suite available from Diamond Light Source. The 2D scattering patterns were integrated using azimuthal integration to generate the 1D scattering patterns.

Synchrotron SAXS measurements presented in Figures 1D,E and S3 were performed at the 12-ID-B beamline of the Advanced Photon Source at Argonne National Laboratory. The wavelength was set at 0.9322 Å during the measurements. Samples were measured using a sample camera distance corresponding to an accessible momentum transfer vector range of 0.025 nm^–1^ < *q* = (4π/λ) sin(θ/2) < 5 nm^–1^. Scattered X-ray intensities were measured by using a Pilatus 2 M (DECTRIS Ltd.) detector. Polymer microgels formed from pNIPMAM with *d*_hyd_ (25 °C) = 355 nm were prepared at 20 mg mL^–1^ in water and loaded into capillaries (with diameter Ø = 2 mm) and sealed properly to prevent the loss of water. The measurement was carried out with 3 heating–cooling cycles from 25 to 55 °C. No significant difference was found between different cycles. Additionally, a blank water sample was measured 3 times at both 25 and 55 °C.

### Dynamic Light Scattering (DLS)

Samples were prepared by dissolving freeze-dried pNIPAM/pNIPMAM polymer in Milli-Q water at 5 mg mL^–1^. The *Z*-average hydrodynamic size and Polydispersity Index (PDI) were measured using a Malvern Nanoseries Zetasizer with laser wavelength 633 nm (Malvern Instruments Ltd., Malvern, U.K.). A volume of 1 mL was measured across the relevant temperature range in 1 °C increments with 300 s equilibration time at each temperature. Three measurements were acquired at each temperature and an average with standard deviation determined. For pNIPMAM microgels, the swollen and collapsed sizes were evaluated as the average size measured from 20 to 25 and 50 to 55 °C, respectively. The same analysis was performed at the pNIPMAM concentrations of 1 mg mL^–1^ (Table S1). In general, collapsed *d*_hyd_ was unchanged compared to samples prepared at 5 mg mL^–1^ (Table 2). Small deviations (<20 nm) in swollen *d*_hyd_ were observed between the two sample concentrations. PDI was lower for most samples prepared at 5 mg mL^–1^.

**Table 2:** Colloidal Characterization of pNIPMAM Microgels in the Swollen and Collapsed States^a^

sample	*d*_hyd_^swoll^ (nm)	PDI^swoll^	ZP^swoll^ (mV)	*d*_hyd_^coll^ (nm)	PDI^coll^	ZP^coll^ (mV)	LCST (°C)^b^
SDS0	**1390**^b^	**0.62**^c^	–6	650	0.20	–23	42.5 ± 0.3
SDS1^d^	780	0.30	–6	440	0.20	–30	43.2 ± 0.5
SDS2	560	0.28	–7	239	0.04	–33	42.0 ± 0.3
SDS3	310	0.07	–6	166	0.11	–32	42.0 ± 0.3
SDS4	157	0.08	–6	71	0.05	–23	42.2 ± 0.1
BIS0	**29**^c^	**0.49**^c^	–8	141	0.02	–27	39.7 ± 0.5
BIS1^d^	780	0.30	–6	440	0.20	–30	43.2 ± 0.5
BIS2	850	0.17	–10	480	0.20	–30	43.2 ± 0.5
BIS3	760	0.14	–12	470	0.24	–33	43.5 ± 0.3
BIS4	706	0.15	–20	470	0.16	–35	46.2 ± 0.1
KPS0^e^	870	0.20	–3	485	0.15	–18^d^	N/A
KPS1	708	0.14	–4	425	0.23	–27	42.0 ± 1.0
KPS2	640	0.14	–4	327	0.20	–32	44.2 ± 0.1
KPS3	885	0.25	–6	402	0.02	–34	44.5 ± 0.3
KPS4^d^	780	0.30	–6	440	0.20	–30	43.2 ± 0.5
KPS5	**850**^b^	**0.37**^b^	–6	440	0.23	–30	46.2 ± 0.5
MG_OPT_^f^	104	0.1	–3	49	0.06	–20	43.0 ± 1.0

a For characterization of *d*_hyd_, all suspensions were measured at 5 mg mL^–1^ and for ZP at 1 mg mL^–1^ (pH 8–8.5). DLS analysis was also performed at 1 mg mL^–1^, and the values obtained were very similar (see Table S1). *d*_hyd_ and PDI are an average of values measured between 20 and 25 °C for the swollen state and between 50 and 55 °C for the collapsed state. All samples were equilibrated for 300 s at the temperature prior to measurement.

b LCST interpreted as maximum rate of change in viscosity with respect to temperature measured using rotational rheology.

c For fields in bold PDI > 0.30, so the *d*_hyd_ value should be viewed with caution.

d SDS1, BIS1, and KPS4 are the same suspension.

e For KPS0, only, the pH was 6.0; otherwise, the pH was ∼7. pH was also found to be insensitive to temperature.

f MG_OPT_ is an optimized microgel formulation based on results obtained from SDS, BIS, and KPS series.

### Zeta Potential and pH

Samples were prepared by dissolving freeze-dried pNIPMAM polymer in Milli-Q water at 1 mg mL^–1^. The zeta potential (*ZP*) of polymer microgel solutions was measured using a Malvern Nanoseries Zetasizer with laser wavelength 633 nm (Malvern Instruments Ltd., Malvern, U.K.). The temperature was set to 25 or 50 °C with 300 s equilibration time at each temperature prior to measurement. Three measurements were acquired at each temperature and an average with standard deviation determined. The pH of the same samples was measured at room temperature and 50 °C. For measurements at 50 °C, samples were equilibrated in a water bath set to 50 °C for 1 h prior to measurement and the measurement was taken while in the water bath. Three measurements were acquired at each temperature and an average with standard deviation reported.

### Rheology

Samples were prepared by dissolving freeze-dried pNIPMAM polymer in Milli-Q water at 50 mg mL^–1^. This was done by gentle vortexing followed by leaving samples for 24 h at room temperature until homogeneous suspensions were obtained with no bubbles. The rheology was performed on a MCR301 rheometer (Anton Paar), using a double gap geometry. 3.8 mL of sample was carefully pipetted onto the bottom plate, and subsequently, the top rheometer plate was lowered slowly to minimize suspension disruption for each measurement. Prior to each day of measurements, the rheometer’s motor was calibrated, and responses were adjusted to those of the viscosity of pure Milli-Q grade H_2_O. For shear rate dependence, samples were measured across a shear rate range of γ̇ = 0.1–1000 s^–1^, at 25 and 50 °C, being held at each shear rate until a stable reading was reported by the instrument. Temperature ramps were measured at a constant shear rate of γ̇ = 10 s^–1^ across a temperature range of 25–50 °C at a  °C min^–1^. For oscillatory temperature ramp, samples were measured at a strain rate, γ of 1% and an angular frequency of 10 rad s^–1^ across a temperature range of 25–50 °C at a rate of °C min^–1^. Prior to all measurements samples were equilibrated for 300 s. All measurements were repeated twice, and average and error between these presented.

### Atomic Force Microscopy

AFM images were acquired in amplitude modulation mode in air and DI water using a commercial AFM instrument (Asylum Research MFP-3D). Imaging in air was performed with an NCH probe (*Nanosensors,* nominal spring constant 42 N m^–1^). Imaging in DI water was undertaken with an SNL C probe (Bruker, nominal spring constant 0.24 N m^–1^) as a function of temperature (Asylum Research BioHeater and fluid cell; quoted 0.02 °C precision and 0.1 °C accuracy with <0.1 °C overshoot). For imaging in the dry state, microgel suspensions (100 μL, 0.35 mg mL^–1^) were predried at 50 °C onto glass slides and then imaged in air at room temperature. For imaging in the wet state, coverslips were first cleaned by sonication in isopropanol for 15 min, dried using compressed N_2_, and exposed to UV ozone for 30 min. Prior to deposition of microgel suspensions, the coverslips were functionalized with poly(allylamine hydrochloride) (PAH) solution to increase the hydrophilicity and aid attachment of microgels. PAH solution (100 μL, 0.2 mM) was pipetted onto the clean coverslips, and spin coating was carried out for 30 s at 2500 rpm. Slides were then washed with Milli-Q water to remove excess PAH solution. Microgel suspensions (100 μL, 0.35 mg mL^–1^) were pipetted onto coverslips at room temperature and incubated for 30 min at 50 °C. For analysis in the wet state, the samples were imaged first at 50 °C, *T* > LCST, and subsequently, the temperature was reduced to 35 °C, *T* < LCST, imaged, and returned to 50 °C. Samples were imaged several minutes after temperature stabilization; the BioHeater heating element is a ring surrounding the sample that heats the solution, while the temperature sensor is more centrally located, near the sample.

## Results and Discussion

### Structural Analysis of pNIPAM and pNIPMAM Micro/Nanogels

Aqueous suspensions of pNIPAM and pNIPMAM microgels were prepared using synthetic conditions adapted from Berndt and Richtering,^31^ i.e., by free radical polymerization of NIPMAM at 70 °C in water in the presence of KPS, BIS, and SDS. The presence of a transition for the pNIPMAM suspensions was apparent as a change in appearance from transparent to cloudy at higher temperature (Figure 1A). This figure includes a schematic representation of the accepted view of a thermoresponsive polymer microgel in the swollen state with chains fully hydrated in the extended coil configuration, which transitions to the collapsed globule state with water expelled from the network. The chemical structures of the reactants are shown for reference in Figure 1B. The suspensions were analyzed using dynamic light scattering (DLS) across the temperature range from 20 to 45 °C for pNIPAM and 25 to 55 °C for pNIPMAM, at a concentration of 5 mg mL^–1^ (Figure 1C). As expected, in both cases, the *z*-average hydrodynamic size, *d*_hyd_, decreased with temperature down to close to the “nanogel” range. The LCST was taken as the temperature of maximum change in *d*_hyd_ with respect to temperature, giving values of 33.5 and 43.5 °C for pNIPAM and pNIPMAM, respectively (Figure 1C). The increased LCST for pNIPMAM is thought to arise from steric hindrance induced by the methyl groups that obstructs hydrophobic interactions.^32^ The measured LCST values are consistent with reported DLS, NMR, and small-angle neutron scattering studies.^8,31^

The structural changes associated with the transition were studied for the same pNIPMAM sample using small-angle X-ray scattering (SAXS). Scattering curves were acquired from 25 to 55 °C, at 20 mg mL^–1^, in the *q* range sensitive to structures from 1.3 to 250 nm in size (Figure 1D). The extracted Guinier slope, *n*, provides the mass fractal dimension (−*n* = *d*_m_) that reflects polymer chain packing within the suspended particles. As expected, *d*_m_ was found to be sensitive to the transition (Figure 1E). In the swollen state, the slope decreased progressively with increasing temperature, with *d*_m_ increasing from 1.37 to 1.65 between 25.0 and 37.5 °C. This shows that the swollen particles have mass-fractal structure with an extended network () and can be taken to comprise weakly interacting (physically cross-linked) branched polymers. The increase in *d*_m_ approaching the transition shows an increase in internal entanglement,^33^ suggesting some loss of water, consistent with the small decrease in *d*_hyd_ in this range (Figure 1C).

On further increase in temperature, a sharp decrease of slope was observed to a final *d*_m_ of ∼3.7. This high fractal dimension corresponds to a three-dimensional sphere with a rough surface.^33^ The maximum change was observed at 44.5 °C, very close to the LCST from DLS of 43.5 °C. Above 46–48 °C, the range in which *d*_hyd_ stabilized at 170 nm, the *d*_m_ value stabilized at ∼3.7, showing that the microgels are fully collapsed. A log-normally distributed hard sphere model was fitted to the SAXS response at 55 °C, Figure S3, giving a collapsed particle size, *d*_SAXS_, of 188 ± 13 nm, very close to the *d*_hyd_ value of 170 nm. This agreement may be fortuitous, given the differences in these techniques, as described by Seelenmeyer et al.^18^ Similar slopes/mass fractal dimensions have been reported from SAXS and SANS analysis of pNIPAM and pNIPMAM microgels with values of ∼1.5 in the swollen and ∼4 in the collapsed state.^24,31,34^ To the best of our knowledge, the effect of composition on structural changes across the transition, measured by SAXS, has not been previously reported for pNIPMAM.

### Effect of Reaction Formulation on Physical Properties of pNIPMAM Micro/Nanogels

**Figure 2:**
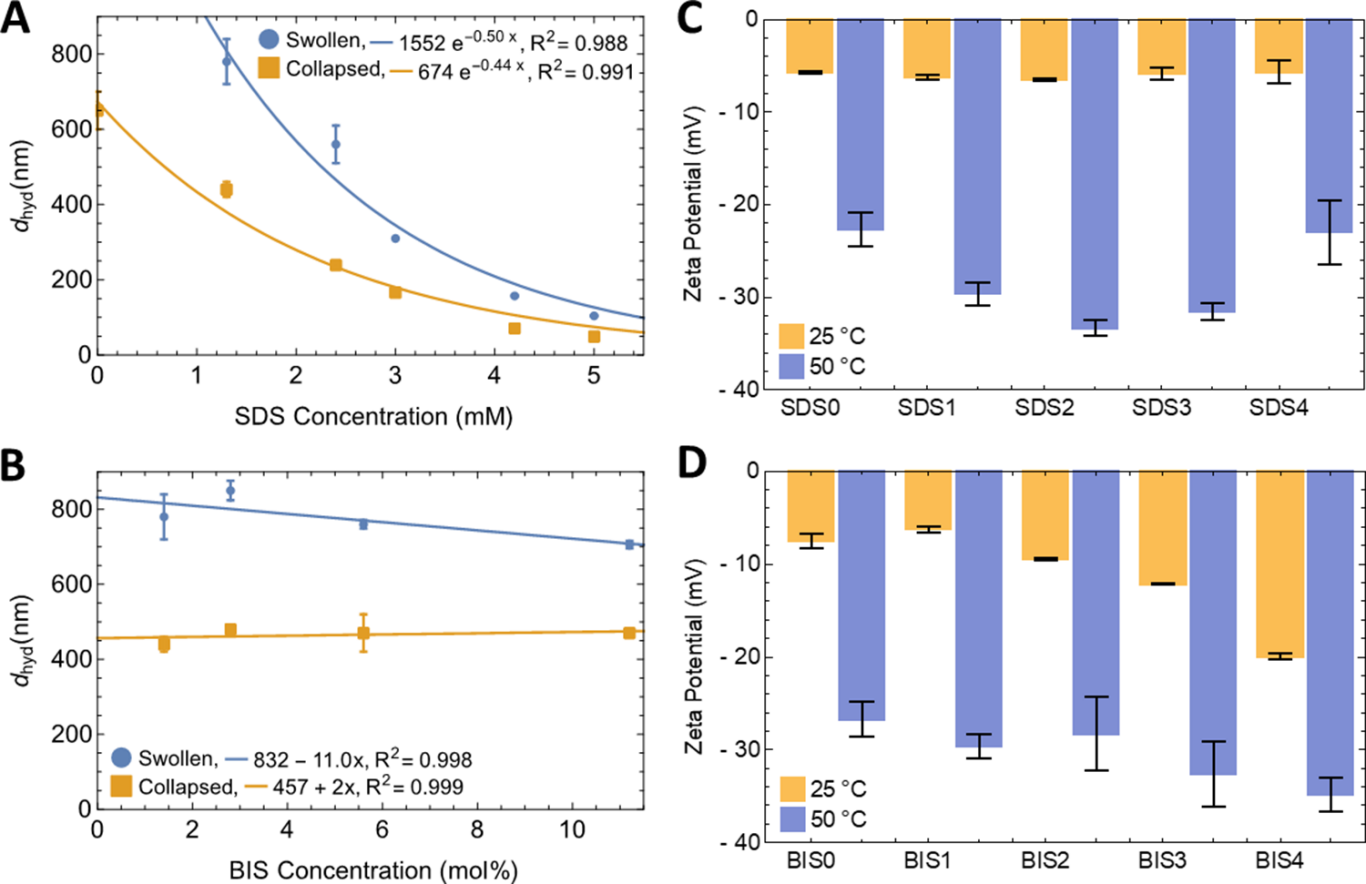
Colloidal characterization for the BIS and SDS series. *d*_hyd_ in the swollen state at 25 °C (blue) and collapsed state at 50 °C (red) state for (A) SDS with an exponential fit included and (B) BIS series with a linear fit. Error bars are the standard deviations for *d*_hyd_ in the 20–25 and 50–55 °C ranges. *d*_hyd_ values with corresponding PDI > 0.3 have been excluded. Zeta potential at the same temperatures for (C) SDS and (D) BIS. Full details are provided in Table 2.

Three series of pNIPMAM microgels were prepared by independently varying the surfactant (SDS), cross-linker (BIS), and initiator (KPS) content to evaluate the effect of synthetic conditions on microgel structure and properties. All compositions are given in Table 1. Colloidal (DLS and zeta potential) and rheological characterization was completed for all suspensions above and below the transition. The concentration range in which the hydrodynamic size is independent of concentration was first determined to be <20 mg mL^–1^ (Figure S4). The *d*_hyd_ and PDI (both from cumulants analysis) and ZP values measured at different concentrations in this range are given in Tables 2 and S1, and *d*_hyd_ and ZP are also shown graphically in Figure 2. SAXS and AFM analyses were undertaken for samples from the SDS and BIS series in the swollen state.

In the collapsed state, at *T* > LCST, all suspensions had reduced particle sizes, low PDI values and relatively strong negative ZP, which is consistent with electrostatic stabilization throughout. In the swollen state, DLS is more challenging; in some cases, the cumulants fit to the data was moderate and gave higher PDI values. In Table 2, swollen state suspensions with PDI > 0.3 are marked in bold; these *d*_hyd_ values should be viewed with caution, and they are not included in Figure 2. It is clear from the table that for all the other suspensions, as compared to the collapsed state: (i) ZP, while still slightly negative, is significantly weaker; (ii) *d*_hyd_ is substantially higher; and (iii) PDI is inconsistent but typically higher. The extended hydrated chain structures, shown by SAXS to be mass-fractal with *d*_m_ ∼ 1, Table 3, are associated with increased *d*_hyd_. We suggest that the lower ZP, when collapsed, is due to charged groups being enveloped by extended polymer chains/the slipping plane being less well-defined, as reported for pNIPAM microgels prepared under surfactant-free conditions.^35^ In the following sections, the effects of surfactant and cross-linker content on the colloidal and structural properties are described in detail.

**Table 3:** SAXS Parameters Obtained for pNIPMAM Microgels for SDS and BIS Series Measured in the Swollen State^a^

sample	SDS (mM)	BIS (mol %)	mass fractal dimension, *d*_m_	Bragg peaks equivalent size (nm)			*R*_g_(nm)	shape factor,^b^*R*_g_*/R*_hyd_
SDS0	0.0	1.4	1.14 ± 0.05	425 ± 14^d^	324 ± 11^d^		196 ± 5	
SDS1^c^	1.3		1.08 ± 0.06	314 ± 11	189 ± 6^d^		182 ± 6	0.47 ± 0.02
SDS2	2.4		1.12 ± 0.05	195 ± 7			163 ± 3	0.58 ± 0.05
SDS3	3.0		1.34 ± 0.02				102 ± 3	0.66 ± 0.03
SDS4	4.2		1.40 ± 0.01	154 ± 5			79 ± 2	1.01 ± 0.02
BIS0	1.3	0.0	0.96 ± 0.07				206 ± 4	
BIS1^c^		1.4	1.08 ± 0.06	314 ± 11	189 ± 6^d^		182 ± 6	0.47 ± 0.02
BIS2		2.8	1.03 ± 0.08	294 ± 10	182 ± 6		211 ± 2	0.50 ± 0.01
BIS3		5.6	1.04 ± 0.08	251 ± 8	165 ± 6		211 ± 2	0.55 ± 0.01
BIS4		11.2	1.00 ± 0.10	239 ± 8	159 ± 5	118 ± 4	213 ± 3	0.60 ± 0.01

a At 25 °C and 20 mg mL^–1^, the SAXS data for all samples are given in Figure S2. The *q* range was set to be sensitive to 3.7–349 nm sizes.

b Value for BIS0 is not included as the DLS response was inconsistent, with high PDI.

c SDS1 and BIS1 are the same suspension.

d Peak weaker but detectable.

### Effect of Surfactant Content on Colloidal Properties and Internal Particle Structure

**Scheme 1:**
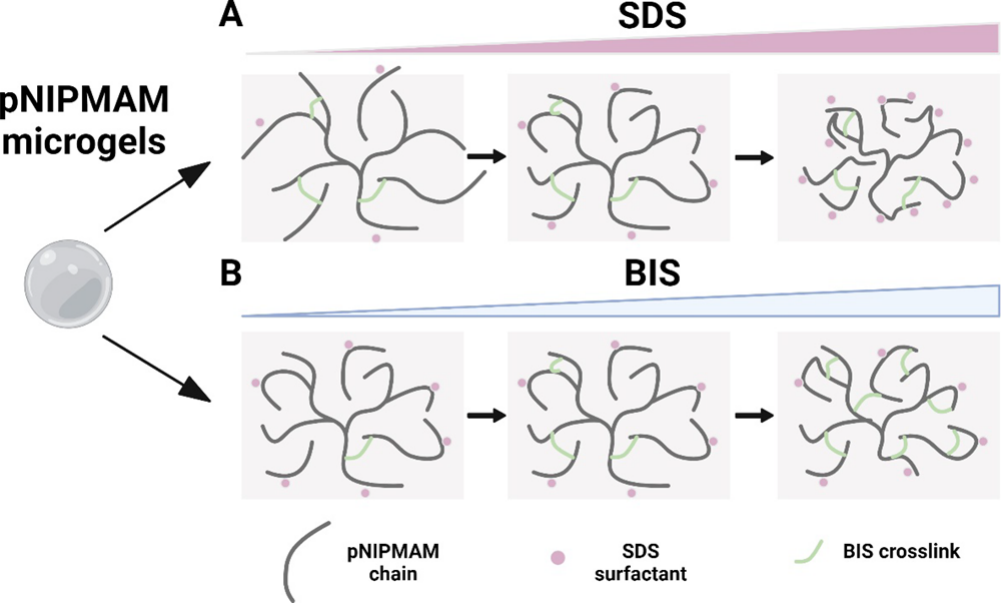
Schematic Showing the Effect of Surfactant and Cross-Linker Composition During Synthesis on the Microgel Network, Shown Here in the Swollen State (A) As SDS content is increased (in a range from 0 mM to below the CMC of SDS), the size decreases and the entanglement increases; (B) as BIS content is increased, there is no change in size or entanglement, but crosslinks increasingly solidify the structure and fold in more of the dangling chains.

In the collapsed state, unlike the other two series, SDS showed a continuous trend in *d*_hyd_ with a progressive decrease in size and PDI with increasing surfactant content (Table 2). As the PDI is consistently low (≤0.30), this change in *d*_hyd_ can be considered a reliable finding; it was found to approximate an exponential decay (Figure 2A). It seems that increasing the surfactant content during synthesis does indeed promote nucleation, as noted above and shown in Scheme 1A. For SDS0, as expected, *d*_hyd_ was the highest measured, and despite the absence of surfactant, the ZP was high, at −23 mV. This shows that negatively charged groups at the surface, originating from the initiator, contribute to surface charge. The increased ZP observed when SDS is included (average of −31 ± 2 mV for SDS1–3) shows that SDS also contributes.

The *d*_hyd_ values were larger in the swollen state than in the collapsed state. For SDS1–4, which retained low *PDI, d*_hyd_ decreased, again, approximately exponentially, with increasing SDS content (Table 2). The Guinier mass fractal dimension was measured by SAXS at 25 °C, and the *d*_m_ values were found to increase with the SDS content. The PDI values suggest three ranges: (i) for the two highest content samples PDI is very low, *d*_hyd_ is the smallest measured, and *d*_m_ was 1.37 ± 0.03; (ii) for the two intermediate content samples, PDI is intermediate, but in the range of unimodal distributions, *d*_hyd_ increased and *d*_m_ was lower at 1.10 ± 0.02; and (iii) at zero content the suspension, while stable, gave inconsistent fluctuating DLS scattering (this stabilized above the transition), the PDI was high so *d*_hyd_ is not interpreted, and *d*_m_ did not change further. The inverse relationship between *d*_m_ and *d*_hyd_ for the SDS containing samples is interesting; it shows a change from a lower extent of persistent entanglement, with more 1D-like disentangled polymer chains, to increased but still relatively weak entanglement at higher content/smaller size.

We suggest that during synthesis, which is at high temperature, growing microgels are “collapsed” and higher surface SDS coverage causes greater intermicrogel electrostatic repulsion compressing the internal structure. This is effectively a “crowding” effect resulting from reduced surface tension, as mentioned earlier. The resulting increased entanglement is retained on cooling into the swollen state, so the size trend remains. Reduced ZP suggests that in the swollen state, the surfactant predominantly resides within the polymer network slightly increasing entanglement (higher *d*_*m*_); however, polymer–solvent interactions are predominant.

Previously von Nessen et al.^25^ reported similar trends *d*_hyd_ for pNIPMAM in the swollen and collapsed states, despite using different KPS and BIS content. Like that study, we also see no significant change in the LCST with SDS concentration. Unlike that study, we find no decrease in LCST at zero SDS content, with an average of 42.4 ± 0.5 °C determined in our case for all five samples. We confirmed the absence of a change in LCST using multiple rheological techniques, see below.

Increasing SDS content was also found to shift the Bragg peaks to smaller size (Table 3). As the SAXS measurements were sensitive to the 3.7–349 nm size range, these features arise from repeat distances between neighboring polymer domains. The shifts indicate changes in internal microgel ordering which we attribute to SDS-induced compression of the internal structure, as suggested above. For SDS0 and SDS1, multiple Bragg peaks were identified, although some of these were weak and poorly defined, perhaps reflecting structural changes in the entanglement between core and extended “brush-like” shells. The absence of multiple Bragg peaks at higher SDS (SDS2–4) could be an effect of a broader size distribution; however the PDI values were low for these samples.

Direct comparison of sizes obtained from DLS and SAXS is difficult given the nature of the techniques.^16,18^ Nevertheless, the radius of gyration, *R*_g_, values were estimated from the partial Guinier regime (Table 3). As they come from the same data, this radius also progressively decreased strongly with SDS content. The shape factor, calculated as *R*_g_*/R*_hyd_ (where the *R*_g_ value was taken from SAXS and *R*_hyd_ from DLS), was found to increase from 0.47 ± 0.02 to 0.66 ± 0.03 at the highest SDS content, which is also consistent with a trend toward more compact structures. For SDS4, *R*_g_*/R*_hyd_ was 1.01 ± 0.02, suggestive of some aggregation of particles at high content; however, this was not observed by DLS.

Although unconventional compared to typical modeling approaches,^16,18,36^ our use of Bragg peak shifts and the changes in *R*_g_*/R*_hyd_ provide strong evidence of a single polymer domain, i.e., the absence of core–shell structure at higher content. In summary, increasing SDS content enables reduction of particle size into the nanorange with increasing entanglement and with no effect on the LCST.

### Effect of Cross-Linker Content on Colloidal Properties and Internal Particle Structure

For the BIS series in the collapsed state, there was no change in *d*_hyd_ or PDI with increasing content, and as PDI ≤ 0.3 throughout, this can again be considered a reliable finding. For BIS1–4, the similarity in size (average 465 ± 17 nm, for BIS1–4) (Figure 2B) suggests that the cross-linker does not directly affect the growth mechanism. For these four samples, the ZP values were also invariant at −31 ± 3 mV (Figure 2D). BIS0 had very low *d*_hyd_, of 141 nm, and PDI, of 0.02, see below.

In the swollen state, as for the SDS series, the *d*_hyd_ values were significantly higher. However, again they did not change (ave. 774 ± 60 nm, for BIS1–4) with BIS content, Figure 2B, as was also the case for *d*_m_ (ave. 1.03 ± 0.04). This confirms, for the first time, that cross-linker content has no significant effect on the packing, which is determined instead by surfactant content. Across the series, the swelling factor, calculated as the ratio of swollen to collapsed spherical hydrodynamic volume, progressively reduced from 5.6 ± 0.5 (BIS1) to 3.4 ± 0.1 (BIS4), in agreement with previous observations for pNIPMAM microgels.^30^ This suggests that the presence of cross-linker limits the extent of collapse by increasing the rigidity of, similarly entangled, polymer networks (Scheme 1B).

Between BIS1 and BIS4, the *R*_g_*/R*_hyd_ values increased from 0.47 ± 0.02 to 0.60 ± 0.01, consistent with marginal progressive increase in compactness and so a reducing fraction of dangling chains. For an ideal compact hard sphere, *R*_g_*/R*_hyd_ is 0.77,^37^ suggesting that even for BIS4, some dangling chains remain at the surface. Additionally, the *R*_g_ value was relatively consistent, demonstrating that BIS content has a weak influence on density and so presumably on the growth mechanism. On the other hand, increasing BIS led to changes in the Bragg peaks, which, unlike SDS, did not decrease in number but, like SDS, did shift to smaller size (Table 3, Figure S2). This also indicates cross-linker content-dependent internal changes in the entangled networks (Scheme 1B). It may be that increasing BIS reduces the size of regions with differing cross-linking density or increases the local gradient in cross-link density. To probe these aspects, we attempted to fit the SAXS data using more sophisticated sphere-based core–shell models.^16,18,31,36^ However, deciphering the exact nature of the structures proved difficult. We suggest that our approach, focusing on the shifts in the Bragg peaks and in *R*_g_*/R*_hyd_, is justified by the consistency of the outcomes, as shown in Table 3.

For the BIS series, as for SDS, the ZP values remained negative, but less strongly so, on swelling. The (swollen) values increased gradually with BIS content, reaching −20 mV for BIS4, as shown in Figure 2D. This change in ZP is moderate, but its progressive nature along the series suggests the trend is real. Negative surface charge could arise from cross-linker groups that reside close to the surface or from initiator/surfactant groups that, as a consequence of increased rigidity, are not fully enveloped within the network in the swollen state. As in the collapsed state, BIS0 was unusual with very high PDI and hence unreliable *d*_hyd_, which we do not interpret. Interestingly SAXS shows that *d*_m_ is almost unchanged for this sample when swollen, despite the loss of monodispersity. This suggests that reducing cross-linker content, even to zero, does not significantly alter entanglement of the swollen particle cores. However, the outer chains do seem to be more disentangled, giving rise to the high PDI.

Most interestingly, the LCST values progressively increased with increasing cross-linker content, Table 2, for all samples including BIS0. This is contrary to previous reports of decreases, for microgels prepared under surfactant-free conditions in which case the LCST was determined using optical density measurements only.^22,30,38^ Our observations for surfactant-containing preparations are confirmed using multiple rheological measurements below. The effect of the cross-linker on LCST in pNIPMAM has not, to the best of our knowledge, been explained. There are however many studies reporting reducing LCST with increasing chain length in thermoresponsive polymers.^39^ These effects were rationalized in terms of an increasing hydrophobicity. We suggest that inclusion of more hydrophilic amide groups (from BIS) increases the LCST because higher temperature is required to disrupt the more plentiful hydrophilic interactions. We have also shown that BIS increases the rigidity of the network, which would restrict chain collapse/water expulsion. The entropy gain is expected to be lower at the transition point in this case, necessitating a higher temperature.

In summary, for pNIPMAM microgels, increasing BIS content did not change microgel size or entanglement but enabled tuning of the LCST due to progressive changes in hydrophilicity and increased microgel rigidity. For pNIPMAM microgels prepared in the presence of surfactants, control over size (but not LCST) is possible through surfactant content, while control over LCST (but not size) is possible through cross-linker content.

### Effect of Initiator Content on pNIPMAM Colloidal Properties

The effect of KPS content was also assessed, and the trends proved to be weaker, but for completeness, the colloidal characterization is included here. In the collapsed state, all reactions that contained initiator produced suspensions with low PDI (≤0.23) and similar sizes (ave. 407 ± 47 nm) to the other series, but with no apparent trend in *d*_hyd_, PDI, or LCST (ave. 44.0 ± 1.6 °C) (Table 2, Figure S6). The KPS series was prepared at the same surfactant concentration (1.3 mM) as SDS1, and the collapsed sizes are similar to that suspension. This indicates that once KPS is present, the size is primarily dictated by surfactant content. ZP was again strongly negative in all cases (Figure S7). In the absence of initiator, for KPS0, *d*_hyd_ was slightly greater at 485 nm and the PDI remained relatively low. The ZP was lower than that for the other suspensions at −18.2 mV, albeit at a lower pH of 6, arising in this case from SDS at the surface. The higher ZP values measured for the rest of the series suggest a further contribution from surface groups originating from the initiator, as was observed for the SDS series. In the swollen state, relatively monodisperse microgels were formed at lower KPS content (up to KSP4), again no trend was apparent in *d*_hyd_ and ZP was weakly negative throughout.

### Optimized pNIPMAM Formulation, MG_OPT_

We have shown that by maximizing SDS and using a KPS concentration in the 1–4 wt % range, microgels of small size and exceptional monodispersity can be obtained. Additionally, by choosing BIS between 1.4 and 2.4 mol %, a biologically appropriate LCST tunable between 39 and 46 °C can be achieved. Finally, an optimized formulation, MG_OPT_, was prepared using 5 mM SDS, 1.8 mol % BIS, and 3.4 wt % KPS (Table 2). High SDS concentration was used to keep the (swollen) size as low as possible for potential bioapplications. Midrange BIS concentration was used to give LCST 5–6 °C above physiological values but below the range for certain cellular apoptosis. The resulting suspensions were found to have *d*_hyd_ 106 nm (*PDI* 0.09) in the swollen and 49 nm (0.01) in the collapsed state, i.e., reaching the nanorange. The phase transition for MG_OPT_ was unchanging (*p* > 0.05) when measured using multiple techniques, with values of 45.0 ± 0.5, 43.0 ± 1.0, and 41.0 ± 2.0 °C obtained from DLS, rotational, and oscillatory rheology, respectively. This shows stability at physiological temperatures and LCST values in the desired range for the smallest monodisperse microgels obtained in this study.

### Atomic Force Microscopy Analysis of the SDS and BIS pNIPMAM Series

**Figure 3:**
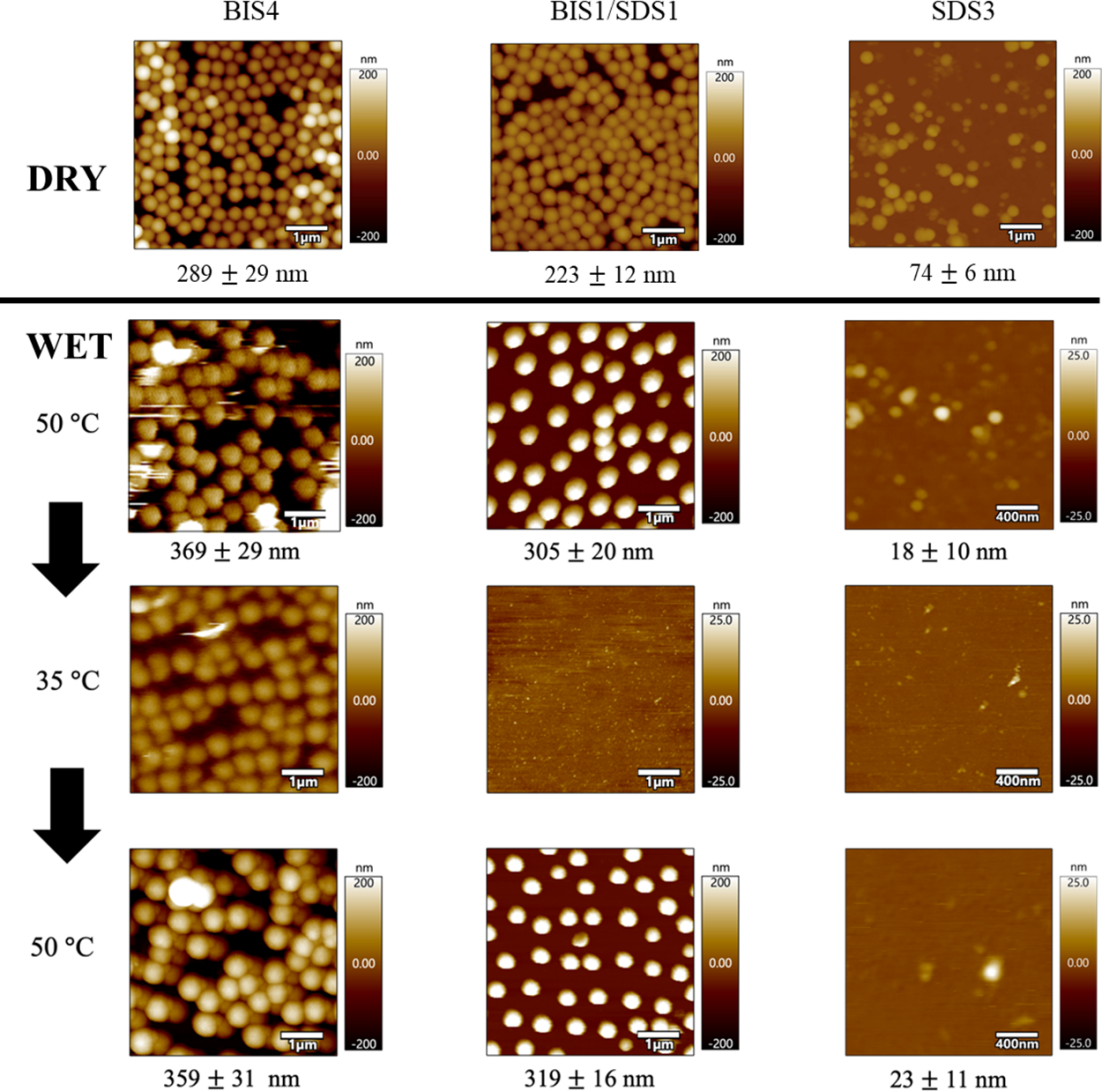
Representative AFM height images of pNIPMAM microgel samples in dry and wet states, BIS4 (5 × 5 μm sizes), SDS1/BIS1 (the same sample, 5 × 5 μm), and SDS3 (1 × 1 μm). Samples (0.35 mg mL^–1^) were deposited onto glass slides and imaged in air at room temperature after incubation at 50 °C for the dry state and for the wet state onto PAH-coated glass coverslips, incubated at 50 °C and imaged in a liquid AFM cell initially at 50 °C (*T* > LCST); subsequently, the temperature was reduced to 35 °C (*T* < LCST), samples were imaged, returned to 50 °C, and imaged again. Average collapsed *d*_AFM_ values were determined as the particle height from several images (the number of particles counted was 35–40 for wet and >10 for dry).

To evaluate the effect of surfactant and cross-linker content on the thermoresponsive nature of the microgels and its impact on morphology and attachment, three representative samples, BIS1 (also labeled SDS1), BIS4, and SDS3, were imaged using AFM in dry and wet states in air and liquid, respectively (Figure 3). In the dry state, microgels are present for all three samples showing that incubation at 50 °C results in particle attachment even in the absence of substrate functionalization (see also Figure S8). The average particle size was determined as particle height from 10 particles for each sample. To assess the thermoresponsive nature of the microgels and their reversibility, microgels prepared on PAH-coated glass coverslips were imaged initially at 50 °C, subsequently below the transition at 35 °C, and finally again at 50 °C in a liquid environment.

Initially, in liquid state AFM measurements for collapsed microgels at 50 °C, particles of defined shape and size were clearly detected for all samples. BIS content was found to have a minimal effect on size (compare SDS1/BIS1 and BIS4), confirming DLS observations. The size decreased with increasing SDS content (SDS1/BIS1 and SDS3), again in agreement with DLS. SDS3 had an unusual high temperature AFM response, although in all other respects, it was a well-behaved suspension. The definition of the particles in the images was less good, the apparent size was far lower than the (collapsed) *d*_hyd_, and the size distribution was seemingly wider. As the entanglement, measured by *d*_m_, is higher, this probably is not due to greater compressibility. It is also noteworthy that for these three samples, the swollen state *ZP* was strongly negative (−30 to −35 mV); electrostatic interactions with the positively charged PAH-coated surface probably helped retain the particles in place. The differences for SDS3 may arise from its smaller size (*d*_hyd_ 166 nm, *d*_AFM_ 18 nm); however, this is a minor point that would require extensive further study.

In the swollen state at 35 °C imaged in liquid, defined microgels were detected only for BIS4 (with a diameter of ∼500 nm inferred from points of contact). While again care should be taken in comparing sizes from the different techniques, this is broadly comparable with the *d*_hyd_ value of 706 nm. No particles were observed for SDS1/BIS1 and SDS3 (both prepared at 1.4 mol % BIS), possibly due to particle detachment from the substrate. In suspension, BIS4 retained a high ZP of −20 mV and microgels were detected, while BIS1/SDS1 and SDS3 both have ZP of −6 mV and microgels were not observed. This and the very similar *d*_hyd_ of BIS1/SDS1 and BIS4 show the importance of electrostatic interactions in AFM detectability, as previously described for charged biopolymers and nanoparticles.^40^ BIS4 detectability may also be favored by the increasingly cross-linked core and reduced number of dangling chains, as indicated by SAXS.

On returning back to 50 °C, microgels with defined shape and the original size were observed for BIS4, and also for SDS1/BIS1. For the latter they are therefore present but not detectable at 35 °C. The AFM results demonstrate good reversibility about the phase transition for pNIPMAM microgels prepared under these conditions, consistent with the observations from temperature-cycled DLS (Figure S9). They also identify the role of electrostatic interactions in retaining particles in place. Similar temperature-dependent AFM detectability was shown for ultralow cross-linked pNIPAM microgels using liquid mode AFM by Schulte et al.^41^ In this case, nondetectability, when swollen, was shown to arise from increased compressibility rather than detachment. In summary, the AFM study supports the picture shown in Scheme 1, of reducing size with SDS but not with BIS content, and suggests increasing rigidity with increasing BIS content.

### Rheological Properties of pNIPMAM Microgels

Rheological analysis was undertaken first to support the findings of the effects of SDS and BIS content on the LCST. Second, we were interested in evaluating how the internal microgel properties of the three series impacted applications that depend on flow, e.g., injectability or extrudability. For this reason, a higher (applications relevant) concentration, of 50 mg mL^–1^, was used. Nevertheless, this is low for rheology, necessitating the use of a double gap geometry which provides high sensitivity.

**Figure 4:**
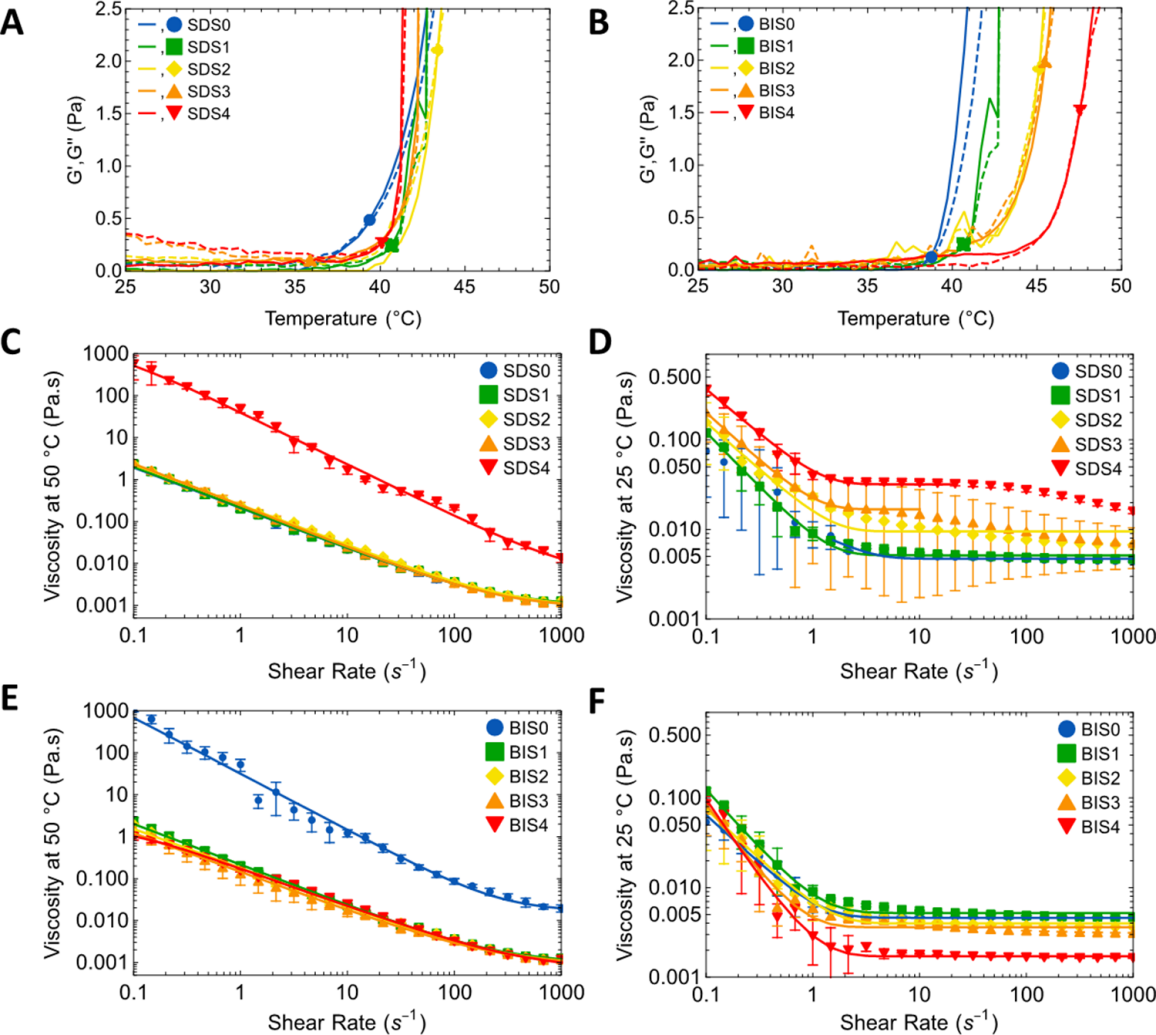
Storage (*G*′, solid lines) and loss (*G*″, dashed lines) modulus measured in oscillatory mode for (A) SDS and (B) BIS. *G*′/*G*″ intersection temperatures are marked with data points. Angular frequency was 10 rad s^–1^, and measurements were undertaken from 25 to 50 °C with 0.5 °C increments, at 50 mg mL^–1^. Average of two or three measurements are reported with the range represented by error bars. (C–F) Dependence on viscosity on shear rate (0.1–1000 s^–1^) for collapsed (left) and swollen (right) pNIPMAM microgels in suspension (at 50 mg mL^–1^) measured in rotational mode. Samples were prepared using 0–4.2 mM SDS (C,D) and 0.0–11.2 mol % BIS (E,F). Each suspension was measured twice, and the average and range (error bar) plotted. Data at 25 and 50 °C were fitted (solid lines) to the Blau and Carreau models, respectively.

The LCST trends, Table 2, were confirmed by measuring the dependence of viscosity on temperature between 25 and 50 °C in oscillatory mode at constant angular frequency, Figure 4, A,B, and also in rotational mode at constant shear of 10 s^–1^ (Figure S10). For the SDS and KPS series, there were no significant changes/trends in the values. For SDS average values, 39.3 ± 1.8 and 42.4 ± 0.5 °C were found for oscillatory and rotational modes, respectively. For BIS, an increase in LCST with content was observed in both modes; the oscillatory mode values progressively increased from ∼38.3 ± 0.5 °C for BIS0 up to ∼47.5 ± 0.1 °C for BIS4, which are very similar to the DLS values. The values (for BIS0 and BIS4) are statistically different (*p*-value < 0.001). A similar increase from ∼39.7 ± 0.5 °C up to ∼46.2 ± 0.1 °C was observed in rotational mode (Table S2). These observations confirm the DLS findings of potentially useful control over LCST, through the cross-linker content only, contrary to the previous reports.^25^

The dependence of viscosity on shear rate (γ̇) was then evaluated for the swollen and collapsed state as a preliminary evaluation of injectability/extrudability. Note that as the concentration used for all measurements was 50 mg mL^–1^. With decreasing size, down the SDS series, the particle concentration increases. Data for the SDS and BIS series are shown in Figure 4C–F and for KPS in Figure S11. The data were successfully fitted using the Blau, Equation S1, and Carreau, Equation S2, approaches to model the dynamic behavior at temperatures below and above the phase transition, respectively. The outcomes of that analysis support the qualitative interpretation provided here and are discussed in SI (Tables S3 and S4).

In the collapsed state, the viscosity of the suspensions was high, Figure 4,C,E, indicative of network-like aggregation due to attractive interparticle interactions.^42^ Shear thinning was observed across the range up to extremely high shear rates, close to the highest γ̇ tested, of ∼1000 s^–1^, indicating that a single process is involved. We suggest there is progressive intermicrogel disengagement under shear. In some cases, there may be a Newtonian regime emerging at high shear rate (e.g., for BIS0 at γ̇ ≳ 800 s^–1^), associated possibly with maximal disengagement. Interestingly, for most suspensions (SDS0–3, BIS1–4), the shear rate-dependent viscosity was remarkably similar, demonstrating very similar responses for microgels prepared under most synthetic conditions. Clearly in the collapsed state, the viscosity is not sensitive to the differences in internal structure apparent from the SAXS and implied by the AFM analysis. SDS4 and BIS0 were unusual in that they also showed shear thinning but with significantly higher and very similar viscosity. These two suspensions had by far the lowest *d*_hyd_. Hence, we ascribe increased η to higher particle concentration in the suspension. This is not a progressive concentration-dependent effect; instead, it seems to have a relatively abrupt onset as size falls into the nanorange/particle numbers increase.

In the swollen state, the viscosity was far lower, Figure 4, D,F, as expected for more deformable particles. All samples showed shear thinning up to γ̇ = 1 s^–1^. Unlike the collapsed state, there were significant systematic changes in viscosity with content for the different series, reflecting internal changes in the particles. We again ascribe the shear thinning, at γ̇ < 10 s^–1^, to intermicrogel disengagement, probably of freely floating chains on the swollen particles. At higher shear rates (γ̇ 10–80 s^–1^), the samples showed Newtonian fluid characteristics, with viscosity independent of shear for the maximally disengaged but still cohesive, particles. In some cases, this behavior persisted up to the maximum shear rate. No significant changes in *d*_hyd_ or *PDI* were observed after shear rate measurements for the samples from all three series, apart from SDS0 (Table S5), suggesting good recovery/minimal damage and possibilities downstream in flow processing for biomedical applications. SDS0 showed some change in *d*_hyd_ and *PDI* after shear, this is perhaps not surprising for this highly extended/minimally cohesive microgel suspension.

For the SDS series in the swollen state, the higher surfactant content samples (SDS2–4) showed a clear progressive increase in viscosity with increasing content. However, the *R*_g_*/R*_hyd_ values showed increasing compactness along the series and *d*_m_ increased, which predict decreased viscosity. We suggest that the observed increase is due to progressively increasing numbers of intermicrogel interactions (higher microgel concentration), rather than any internal changes. Interestingly, for the two smallest, highest viscosity samples (SDS3–4), shear thinning behavior was observed at a high shear above the plateau. This is probably due to some additional deformation driven by the increasingly numerous interparticle interactions.

For the BIS series, in the swollen state, a Newtonian regime was again observed extending to high shear. The viscosity decreased weakly with content for BIS0–3, for which the values were comparable to those of the low SDS content samples. For BIS4, the Newtonian plateau was achieved at a similar shear, and the viscosity was significantly lower than for BIS0–3. As compared to SDS, this series had higher, unchanging, *d*_hyd_ values and hence unchanging particle concentration, and also unchanging *d*_*m*_ values. We suggest that decreasing viscosity is due to increasingly cross-linked cores, Scheme 1B, resulting in a smaller fraction of dangling chains and so reduced frictional interaction with the shearing solvent. This view is supported by the observations, noted above for BIS1–4, of progressively increasing *R*_g_*/R*_hyd_ (increasing compactness) and progressively decreasing swelling factor (increasing rigidity limiting collapse).

In summary, in the swollen state, the typical temperature range for advanced manufacturing, the correlation between the rheological properties and the composition and size is weak. However, for BIS, there are two, and for SDS three, shear regimes apparent. Depending on the application, the exact shear rate of the thinning to Newtonian transition, at the temperature and concentration used, may impact performance. This study provides preliminary guidelines for designing microgels for such applications.

## Conclusions

We have shown that by controlling the surfactant content, it is possible to suppress the swollen and collapsed microgel size, and this is accompanied by increased internal entanglement. Unlike previous reports,^25^ we observed no dependence of LCST on the presence or content of SDS, and this was confirmed by additional techniques. On increasing BIS content, there was no change in size, but an increase in the LCST was observed. This suggests that the cross-linker groups are involved in hydrogen bonding with the solvent molecules, and increasingly high temperatures are required to disrupt these interactions. This finding was again contrary to previous reports,^22,30,38^ and it was also confirmed by additional measurements. While the change in LCST is weak in absolute terms, it is in an excellent range for use in thermoresponsive materials/devices that use physiological temperature as the resting/stimulus-off state and *c.* 41–46 °C (the range for thermal perturbation of cells without apoptosis)^43^ for stimulus-on. Good evidence was obtained supporting the expected increased rigidity with increasing BIS content. It was found that increasing KPS content had a minimal effect on *d*_hyd_ and *PDI* or on the LCST. Optimized conditions were identified for the preparation of stable pNIPMAM nanogel suspensions with LCST slightly above physiological temperature; these are potentially useful thermoresponsive units for bioapplications. We note that the interaction of proteins with the microgel surfaces (which may alter LCST and the protein conformation^44^) will be significantly different under physiological conditions than for pNIPAM, due to the inherent phase transition temperature and differences in hydrophobicity/H-bonding/local rigidity.

## Supporting Information Available

DLS data used in the interpretation of LCST, fittings for trends in size, and additional data to demonstrate reversibility in size across the phase transition and after rheological analysis; DLS data supporting purification of microgels by dialysis; evaluation of concentration dependence of DLS response; SAXS fitting for microgels at 55 °C and full SAXS scattering curves obtained at 25 °C for SDS and BIS series; AFM images measured in the dry state; details of rheological analysis (Blau and Carreau models) including tabulated data for parameters for each model; and oscillatory and rotational data and LCST values obtained using each technique (PDF)
