# Supplementary material for: Thermoresponsiveness Across the Physiologically Accessible Range: Effect of Surfactant, Cross-Linker, and Initiator Content on Size, Structure, and Transition Temperature of Poly(N-isopropylmethacrylamide) Microgels
Source: ACS Omega. 2024 Aug 12;9(34):36185–97. doi: 10.1021/acsomega.4c02115 (PMC11360016; doi:10.1021/acsomega.4c02115)
Supplement: Supplementary file 1 — ao4c02115_si_001.pdf [file ao4c02115_si_001.pdf]

Thermoresponsiveness across the physiologically accessible range: Effect of surfactant, crosslinker and initiator content on size, structure and transition temperature of Poly(N-isopropylmethacrylamide) microgels – Supporting Information

Danielle Winning[1], Jacek K. Wychowaniec[1, 2*], Bing Wu[3], Andreas Heise[4], Brian J. Rodriguez[5,6], Dermot F. Brougham[1,*]

[1]School of Chemistry, University College Dublin, Belfield, Dublin 4, Ireland

[2]AO Research Institute Davos, Clavadelerstrasse 8, 7270, Davos, Switzerland

[3]Dutch-Belgian Beamline (DUBBLE), European Synchrotron Radiation Facility (ESRF), 71 Avenue Des Martyrs, CS 40220, Grenoble, 38043, France

[4]Department of Chemistry, Royal College of Surgeons in Ireland, Dublin, Ireland

[5]Conway Institute of Biomolecular and Biomedical Research, University College Dublin, Belfield, Dublin 4, Ireland

[6]School of Physics, University College Dublin, Belfield, Dublin 4, Ireland

* Corresponding authors:

J.K.W. Phone: (+41)0779238956;

e-mail: jacek.wychowaniec@aofoundation.org

D.F.B. Phone: (+353)017162077;

e-mail: dermot.brougham@ucd.ie

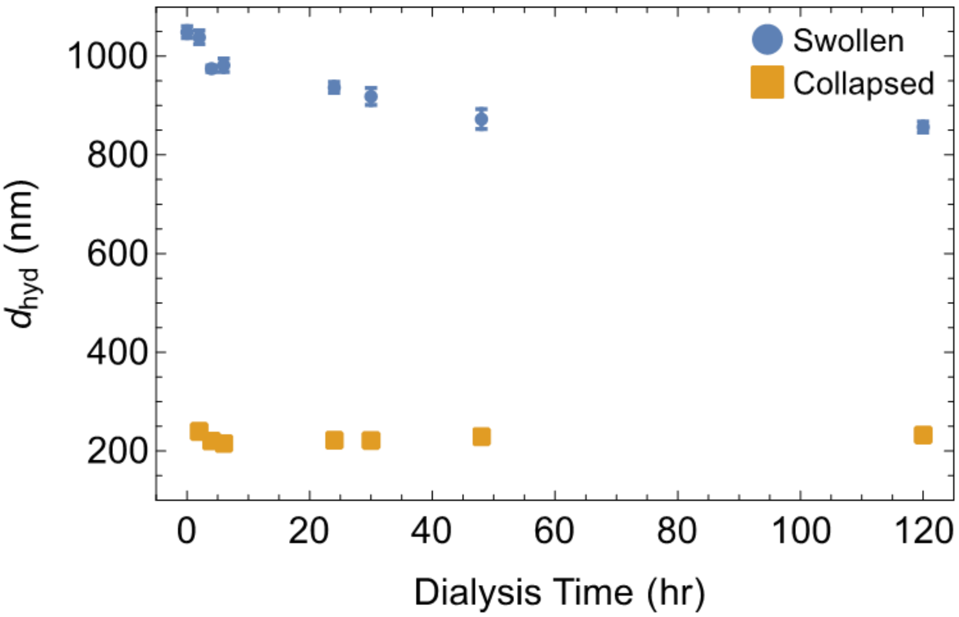

Figure S1: *Progress of dialysis measured by DLS at 25 °C (swollen) and 50 °C (collapsed) for microgel suspensions, prepared using the reactants ratios of SDS1/BIS1 (1.3 mM SDS, 1.4 mol% BIS and 3.4 wt% KPS). Error bars (std dev of 3 successive measurements) are included for all points but are smaller than the data mark size in some cases. Note that at t =0 the PDI > 0.3, so this data is not included.*

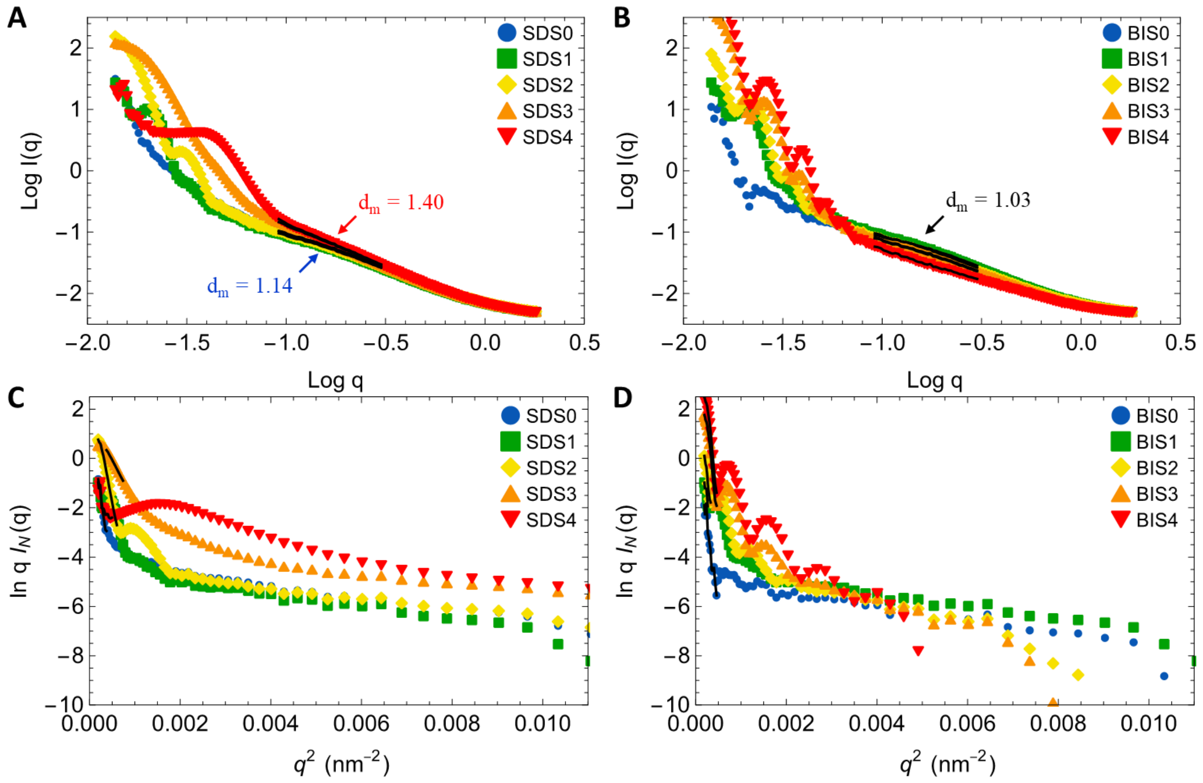

Figure S2: *SAXS patterns obtained for pNIPMAM microgels under varying synthetic conditions: SDS0-4 (0 to 4.2 mM SDS)* ***(A, C)*** *and BIS0-4 (0.0 to 11.2 mol% BIS)* ***(B, D)*** *in log I(q) vs log q n* ***(A, B)*** *and in ln q I(q) vs* q^2 *for low q* ***(C, D)*** *representation. In* ***A*** *and* ***B*** *linear fits were applied (black lines) to extract Guinier's fractal dimension, n, which is summarized in* ***Table 2*** *in the paper. In* ***C*** *and* ***D*** *linear fits were applied (black lines) to extract radius of gyration,* R_g*, values of which are also given in* ***Table 2****. All samples were measured at 20 mg* mL^{-1} *and at 25 °C.*

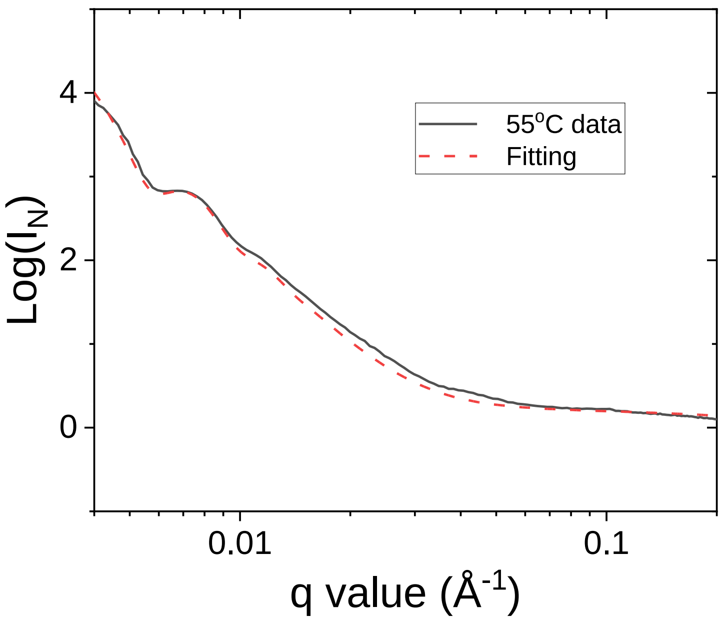

Figure S3: *Fitting of SAXS curve for pNIPMAM microgel at 55 °C. The fitting was performed using SASFit software. The scattering profile of the system is fitted with a combination of hard spheric model and Ornstein-Zernike model, as frequently used in the other similar systems.*[1]

(1) Suzuki, D.; Nagase, Y.; Kureha, T.; Sato, T. Internal Structures of Thermosensitive Hybrid Microgels Investigated by Means of Small-Angle X-ray Scattering. *The Journal of Physical Chemistry B* **2014**, *118* (8), 2194-2204. DOI: 10.1021/jp410983x. Oberdisse, J.; Hellweg, T. Recent advances in stimuli-responsive core-shell microgel particles: synthesis, characterisation, and applications. *Colloid and Polymer Science* **2020**, *298* (7), 921-935. DOI: 10.1007/s00396-020-04629-0.

***Table S1**: Colloidal characterization of pNIPMAM microgels in the swollen and collapsed states at 1 mg mL^{-1} (pH 8-8.5). The d_{hyd} and PDI are an average of values measured between 20 and 25 °C for the swollen state and between 50 and 55 °C for the collapsed. All samples were equilibrated for 300 s at temperature prior to measurement.*

**Sample**	**d_{hyd} ^{swoll} (nm)**	**PDI ^{swoll}**	**ZP ^{swoll} (mV)**	**d_{hyd} ^{coll} (nm)**	**PDI ^{coll}**	**ZP ^{coll} (mV)**
SDS0	**1380**	**0.47**	-6	660	0.21	-23
SDS1	790	0.25	-6	410	0.20	-30
SDS2	580	0.29	-7	250	0.07	-33
SDS3	350	0.10	-6	163	0.06	-32
SDS4	179	0.12	-6	68	0.08	-23
BIS0	**34**	**0.52**	-8	172	0.06	-27
BIS1	790	0.25	-6	410	0.20	-30
BIS2	830	0.18	-10	500	0.22	-28
BIS3	750	0.19	-12	**530**	**0.31**	-33
BIS4	720	0.17	-20	500	0.21	-35
KPS0	820	0.18	-3	480	0.15	-18
KPS1	830	0.18	-4	435	0.23	-27
KPS2	700	0.18	-4	300	0.20	-32
KPS3	**850**	**0.42**	-6	412	0.06	-34
KPS4	790	0.25	-6	410	0.20	-30
KPS5	**880**	**0.40**	-6	480	0.23	-30

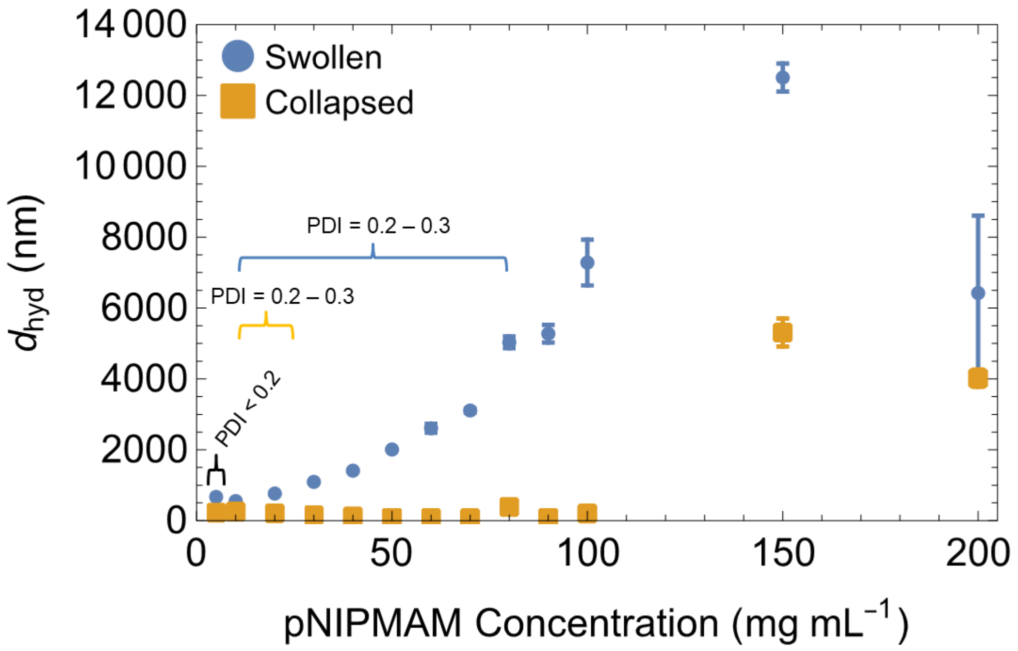

Figure S4*: pNIPMAM microgel concentration dependent DLS analysis recorded at 25 (swollen) and 50 °C (collapsed) for microgel suspensions prepared using the reactants ratios of SDS1/BIS1 (1.3 mM SDS, 1.4 mol% BIS and 3.4 wt% KPS). Error bars (std dev of 3 successive measurements) are included for all points but are smaller than the data mark size in some cases. Note the PDI ranges are as indicated for values ≤ 0.30, for the remaining points PDI > 0.30.*

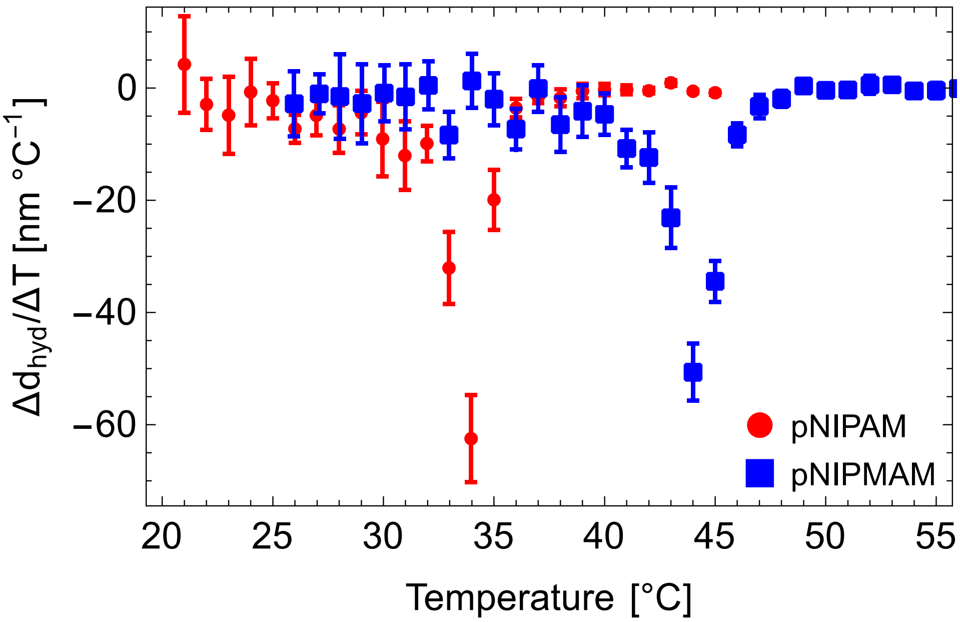

Figure S5: *Rate of change of d_{hyd} with respect to temperature for pNIPAM (red) and pNIPMAM (blue) microgel samples from* ***Figure 1A***. *Samples were prepared using 1.3 mM SDS, 1.4 mol% BIS and 3.4 wt% KPS. Samples were measured by DLS at polymer concentration of 5 mg mL^{-1} and at 1 °C increments across the temperature range, with 300 s equilibration time at each temperature. LCST is interpreted as the maximum rate of change, giving values of 33.5 and 43.5 °C for pNIPAM and pNIPMAM, respectively.*

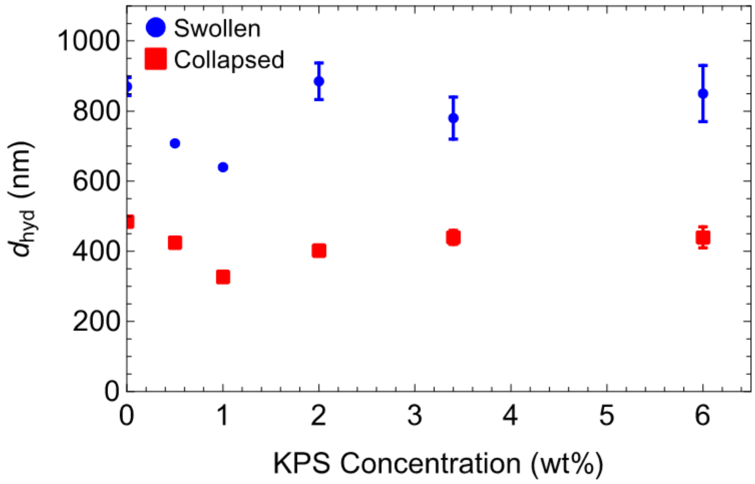

Figure S6: *Plot of d_{hyd} in the swollen (blue) and collapsed (red) state at polymer microgel concentration 5 mg mL^{-1} under varying synthetic conditions of 0 to 6 wt% KPS. Data plotted as an average d_{hyd} across the temperature range of 20 to 25 °C in the swollen state and 50 to 55 °C in the collapsed state. Error bars are a standard deviation across the same temperature ranges.*

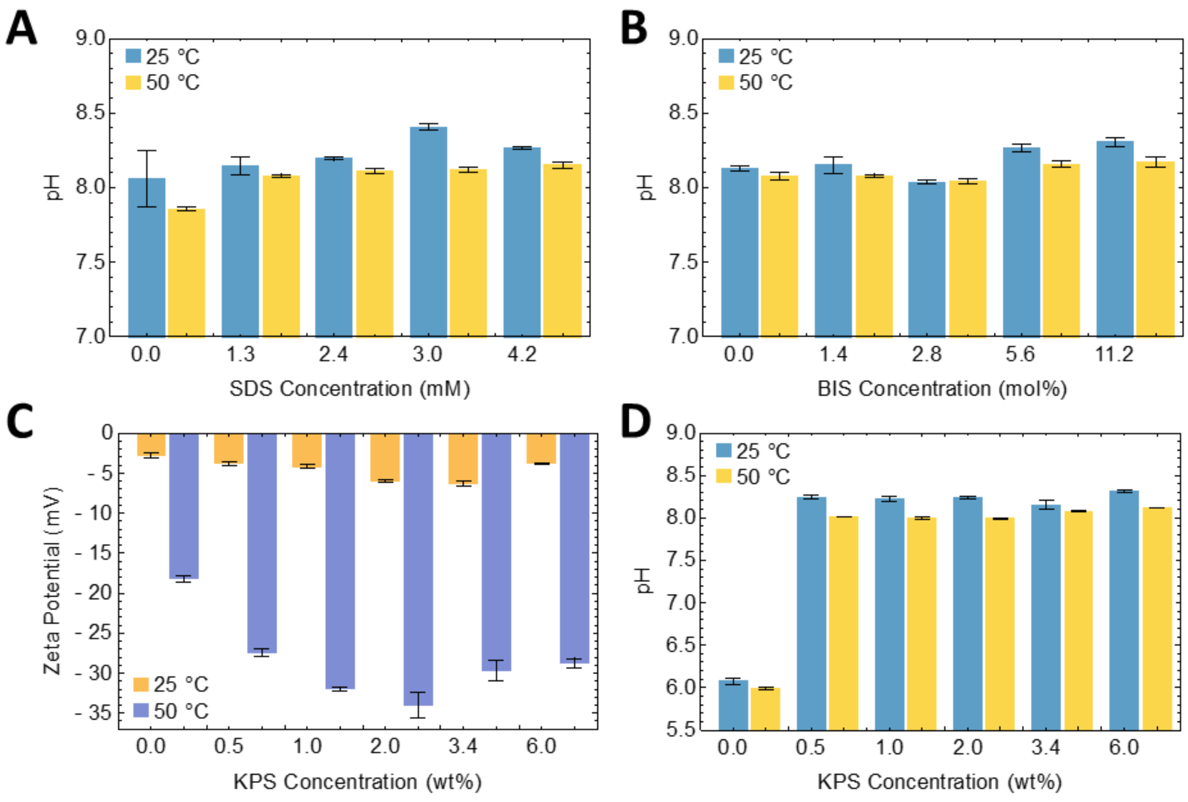

Figure S7*: Zeta potential (****C****) and pH (****A, B, D****) in the swollen (25 °C) and collapsed states (50 °C) for pNIPMAM microgels synthesized at 0.0 to 4.2 mM SDS (****A****), 0.0 to 11.2 mol% BIS (****B****) and 0.0 to 6.0 wt% KPS (****C, D****). All samples were measured three times at 1 mg mL*$^{-1}$ *and an average plotted with error bars as standard deviation.*

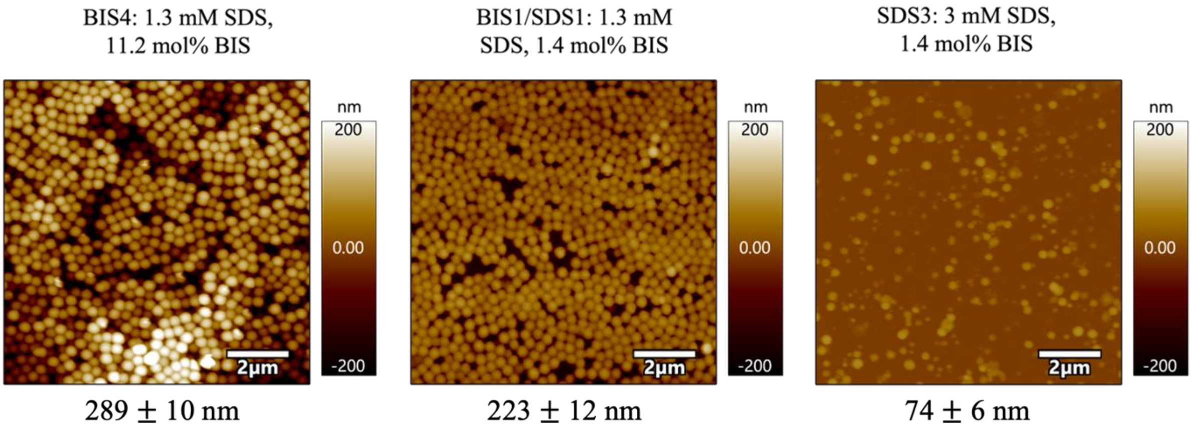

Figure S8: *AFM images of dry pNIPMAM microgel samples BIS4, SDS1/BIS1 and SDS3. Microgel samples (0.035 wt/v%) were deposited onto glass slides and incubated at 50 °C. Average size was determined from 10 particles for each sample. Imaged with NCH probe (Nanosensors) in amplitude modulation mode in air at room temperature by AFM (Asylum Research MFP-3D).*

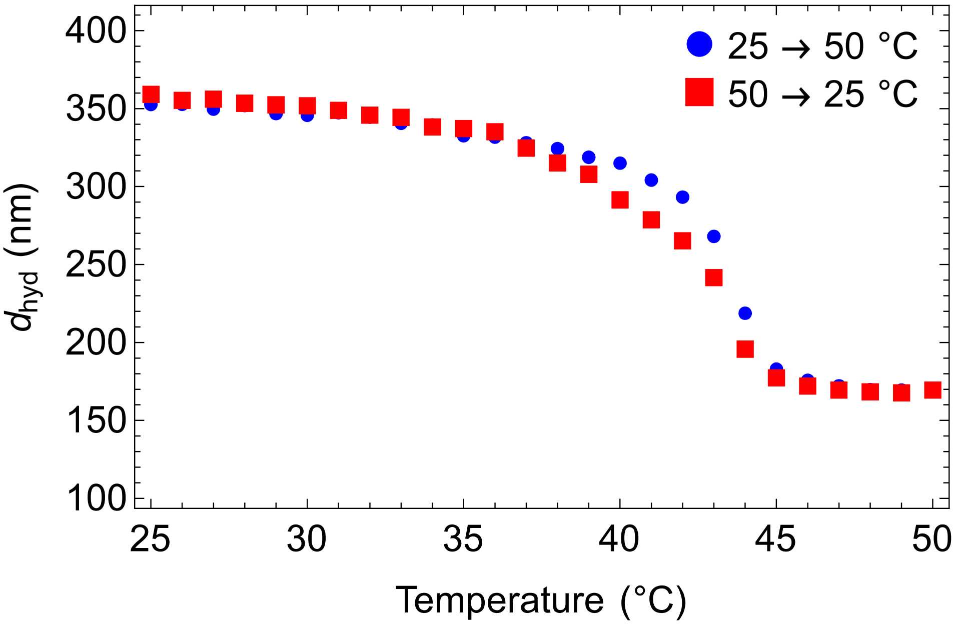

Figure S9*: d_{hyd} as a function of temperature when ramped up from 25 to 50 °C (blue markers) and back down to 25 °C (red markers) for pNIPMAM microgels. This sample is the same as in* ***Figure 1*** *in main paper and* ***Figure S3****. Samples were measured by DLS at polymer concentration of 5 mg mL^{-1} and at 1 °C increments across the temperature range, with 300 s equilibration time at each temperature.*

Table S2: *LCST values interpreted as the maximum rate of change in* d_{hyd} *measured using DLS at 5 mg mL*$^{-1}$*, maximum rate of change in viscosity with respect to temperature measured using rotational rheology and intersection of G' and G" using oscillatory rheology at 50 mg mL*$^{-1}$*. N/A indicates error bars were too large to allow identification of the LCST temperature.*

**Sample**	**LCST (°C)**		
	**DLS**	**Rotational Rheology**	**Oscillatory Rheology**
SDS0	**N/A**	42.5 ± 0.3	38.0 ± 1.0
SDS1[1]	**N/A**	43.2 ± 0.5	40.4 ± 0.4
SDS2	45.0 ± 1.0	42.0 ± 0.3	42.0 ± 0.1
SDS3	44.5 ± 0.5	42.0 ± 0.3	38.0 ± 4.0
SDS4	45.5 ± 0.5	42.2 ± 0.1	38.0 ± 2.0
BIS0	**N/A**	39.7 ± 0.5	38.3 ± 0.5
BIS1[1]	**N/A**	43.2 ± 0.5	40.4 ± 0.3
BIS2	46.0 ± 2.0	43.2 ± 0.5	45.2 ± 0.1
BIS3	45.0 ± 2.0	43.5 ± 0.3	45.4 ± 0.1
BIS4	44.0 ± 1.0	46.2 ± 0.1	47.5 ± 0.1
KPS0	44.0 ± 1.0		
KPS1	45.0 ± 1.0	42.0 ± 1.0	32.2 ± 0.9
KPS2	44.0 ± 3.0	44.2 ± 0.1	41.5 ± 0.4
KPS3	**N/A**	44.5 ± 0.3	43.0 ± 0.2
KPS4[1]	**N/A**	43.2 ± 0.5	40.4 ± 0.3
KPS5	**N/A**	46.2 ± 0.5	43.1 ± 0.3
MG_{OPT}	45.0 ± 0.5	43.0 ± 1.0	41.0 ± 2.0

[1] *SDS1, BIS1 and KPS4 are the same suspension.*

Fitting rheological behavior of microgels

(i) Modelling in the swollen state: Blau model

This model describes a suspension that shows shear thinning behavior at low shear rates and becomes Newtonian at higher shear rates. From this model, we can extract the factor for viscosity (a) and shear rate (b) as well as the coefficient for structure breakdown (c). Parameter ‘a’ is related to the viscosity at low shear and can be interpreted as the viscosity at 0 s^{-1} (η_0). Parameter ‘b’ defines the shear rate at which the viscosity starts to plateau and will be used to describe the point at which the suspension shifts from shear thinning to Newtonian behavior. At this point there is total disentanglement of polymer chains, and they now behave as an ideal fluid. The coefficient for structure breakdown is related to the power law index by 1-n where n is the power law index. The extracted parameters, summarized in ***Table S3***, are an average of the parameters obtained from fits of two data sets collected under the same conditions and are reported within error of the standard deviation between values.

$$\eta = a\left(coth\left(\frac{\dot{\gamma}}{b}\right)\right)^{c} \qquad \textbf{\textit{Equation S1}}$$

(ii) Modelling in the collapsed state: Carreau model

This model describes suspensions showing Newtonian behavior at low and high shear rates, but with shear thinning behavior at intermediate shear rates.

$$\eta = \eta_\infty + \frac{(\eta_0 - \eta_\infty)}{(1+(t\dot{\gamma})^2)^p} \qquad \textbf{\textit{Equation S2}}$$

The parameters η_∞ and η_0 can be extracted using the Carreau model. The parameter, p is related to the power law index (b), **Equation S3**, which denotes whether the material shows shear thinning (b<1), thickening (b>1) or Newtonian (b=1) viscous behavior.

$$p = \frac{1-b}{a}$$ ***Equation S3***

Where ‘a’ is a dimensionless parameter that describes the transition from the zero-shear rate region to the power law region. For the Carreau model, a = 2 and ***Equation S3*** simplifies to **Equation S4**.

$$p = \frac{1-b}{2}$$ ***Equation S4***

Finally the Carreau model allows us to extract a parameter, t, which effectively defines a relaxation time of the studied system.

The extracted parameters, summarized in ***Table S4***, are an average of the parameters obtained from fits of two data sets collected under the same conditions and are reported within error of the standard deviation between values. In general, the fits for both the Blau and Carreau model to the data were good apart from SDS concentrations equal to and greater than 2.4 mM (SDS2, SDS3 and SDS4) at 25 °C, where a second region of shear thinning behavior at intermediate shear rates was observed.

***Table S3:** Extracted parameters from the Blau (**Equation S1**) fits respectively to data in **Figure 3** from main paper for all polymer microgel samples investigated at varying SDS, BIS and KPS concentration. Values are an average of the fits of two data sets, the error shows the range between the two values.*

Sample	Blau at 25 °C		
	a (s^{-1})	b (s^{-1})	c
SDS0[2]	0.0047 ± 0.0001	3.3 ± 0.4	0.6 ± 0.1
SDS1[1]	0.0051 ± 0.0001	1.4 ± 0.5	1.3 ± 0.2
SDS2	0.0091 ± 0.0008	**3.0 ± 2.0**	1.0 ± 0.4
SDS3[3]	**0.0200 ± 0.0100**	1.3 ± 0.5	1.1 ± 0.1
SDS4[4]	0.0318 ± 0.0006	1.2 ± 0.1	1.0 ± 0.1
BIS0	0.0046 ± 0.0001	1.1 ± 0.3	1.1 ± 0.1
BIS1[1]	0.0051 ± 0.0001	1.4 ± 0.5	1.3 ± 0.2
BIS2	0.0034 ± 0.0001	1.4 ± 0.1	1.1 ± 0.2
BIS3	0.0036 ± 0.0001	0.8 ± 0.1	1.5 ± 0.2
BIS4	0.0017 ± 0.0001	1.1 ± 0.2	1.7 ± 0.3
KPS0	0.0041 ± 0.0002	**6.0 ± 3.0**	0.6 ± 0.2
KPS1	0.0158 ± 0.0004	**19.0 ± 7.0**	0.6 ± 0.1
KPS2	0.0135 ± 0.0001	30.1 ± 0.9	0.4 ± 0.1
KPS3	0.0070 ± 0.0001	6.0 ± 1.0	0.5 ± 0.1
KPS4[1]	0.0051 ± 0.0001	1.4 ± 0.5	1.3 ± 0.2
KPS5	0.0059 ± 0.0001	1.7 ± 0.5	0.6 ± 0.1

[1] *SDS1, BIS1 and KPS4 are the same suspension.*
[2] *Fit restricted to 0.681-1000 s^{-1}.*
[3] *Fit restricted to 0.1-10 s^{-1}.*
[4] *Fit restricted to 0.1-21.5 s^{-1}.*

Table S4: *Extracted parameters from the Carreau (**Equation S2**) fits respectively to data in **Figure 3** from main paper for all polymer microgel samples investigated at varying SDS, BIS and KPS concentration. Values are an average of the fits of two data sets within error of the range between the two values.*

Sample	Carreau at 50 °C			
	η_∞ (Pa.s)	η_0 (Pa.s)	t (s)	p
SDS0	0.0009 ± 0.0001	9 ± 1	47.0 ± 0.1	0.49 ± 0.01
SDS1[1]	0.0009 ± 0.0001	9 ± 2	47.4 ± 0.1	0.49 ± 0.01
SDS2	0.0008 ± 0.0001	**8 ± 3**	**37.0 ± 11.0**	0.49 ± 0.01
SDS3	0.0008 ± 0.0001	9 ± 1	**39.0 ± 8.0**	0.50 ± 0.01
SDS4	0.0050 ± 0.0010	**3000 ± 2000**	**27.0 ± 20.0**	0.62 ± 0.01
BIS0	0.0170 ± 0.0030	5670 ± 70	47.0 ± 0.1	0.67 ± 0.01
BIS1[1]	0.0009 ± 0.0001	9 ± 2	47.4 ± 0.1	0.49 ± 0.01
BIS2	0.0008 ± 0.0001	7 ± 1	47 ± 0.1	0.46 ± 0.01
BIS3	0.0008 ± 0.000§	5 ± 2	47.4 ± 0.1	0.46 ± 0.03
BIS4	0.0007 ± 0.0001	2 ± 1	11.0 ± 3.0	0.45 ± 0.01
KPS0	0.0022 ± 0.0002	**49 ± 34**	**25.0 ± 22.0**	0.58 ± 0.02
KPS1	0.0008 ± 0.0001	11 ± 4	47.4 ± 0.1	0.50 ± 0.02
KPS2	0.0008 ± 0.0001	13 ± 2	47.5 ± 0.1	0.50 ± 0.01
KPS3	0.0008 ± 0.0001	16 ± 1	47.1 ± 0.3	0.51 ± 0.01
KPS4[1]	0.0009 ± 0.0001	9 ± 2	47.5 ± 0.1	0.49 ± 0.01
KPS5	0.0008 ± 0.0001	8 ± 3	47.5 ± 0.1	0.46 ± 0.02

[1.] SDS1, BIS1 and KPS4 are the same suspension.
[2.] Values highlighted in bold has significant error and should be viewed with caution.

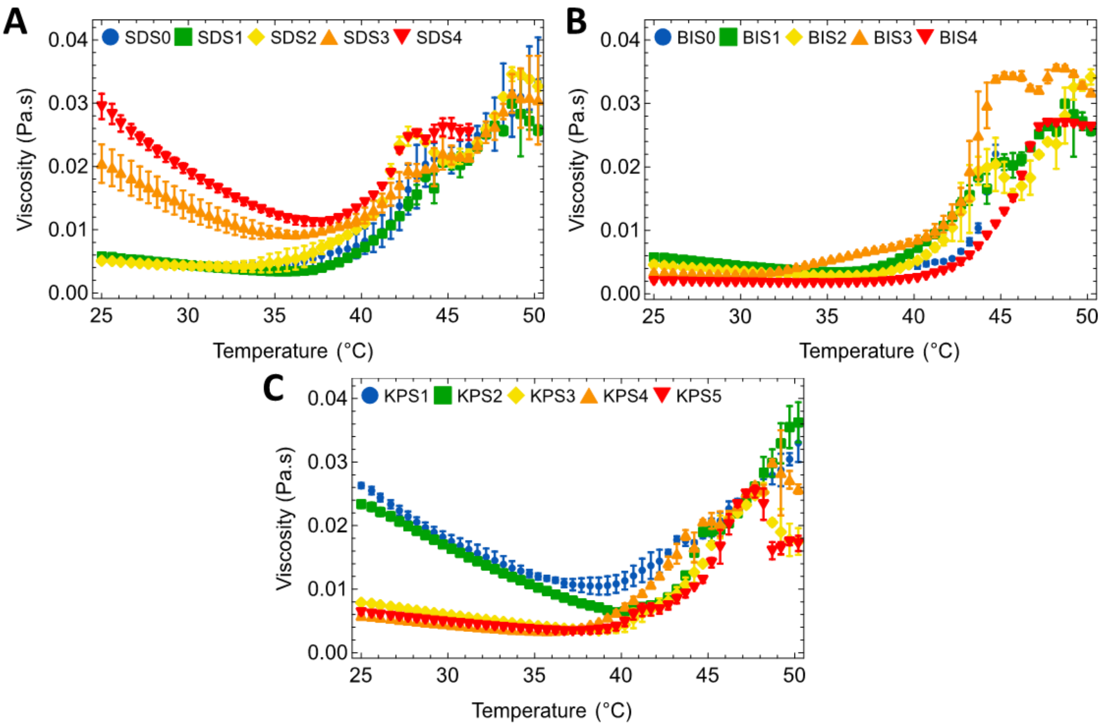

Figure S10: *Temperature dependence of;* ***(A-C)*** *viscosity measured in rotational mode at a shear rate of 10 s^{-1} for* ***(A)*** *SDS,* ***(B)*** *BIS, and* ***(C)*** *KPS series; All measurements were undertaken from 25 to 50 °C with 0.5 °C increments, and at polymer concentration 50 mg mL^{-1}. An average of two measurements have been reported with the range between the two measurements represented by error bars.*

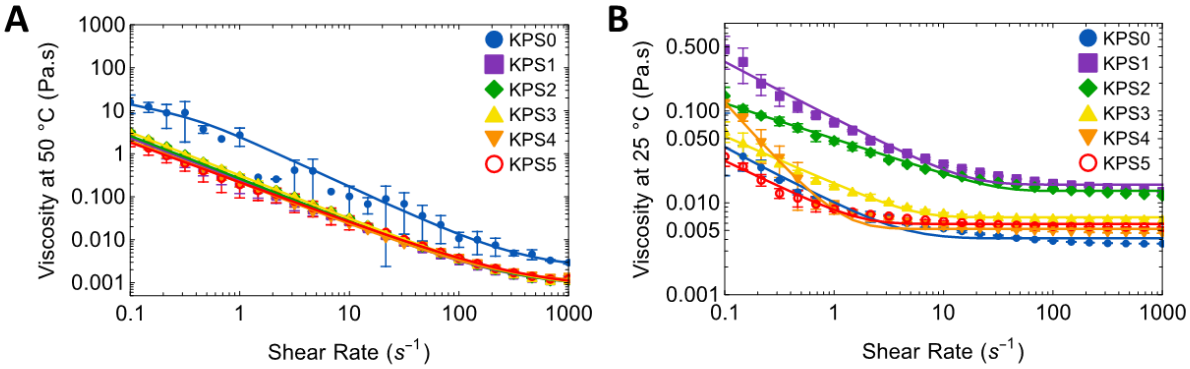

Figure S11*: The dependence on viscosity on shear rate (0.1 – 1000 s*$^{-1}$*) for collapsed (left) and swollen (right) pNIPMAM microgels in suspension (at 50 mg mL*$^{-1}$*) measured using rotational mode. Samples were prepared using 0.0 to 6.0 wt% KPS (****A-B****). Each suspension was measured twice and an average plotted with the range between two measurements represented by error bars. Data at 25 and 50 °C are fitted using the Blau (****Equation S1****) and Carreau (****Equation S2****) models, respectively, the fits are shown as solid lines.*

Table S5: *Change in d_{hyd} measured at 25 °C before and after each rheological analysis for SDS, BIS and KPS series. Values in bold are where PDI > 0.3.*

Sample	Δd_{hyd} (nm) after rheological experiments, re-measured at 25 °C			
	Shear Rate at 25 °C	Shear Rate at 50 °C	Temperature	Oscillatory
SDS0	**160**	**80**	**112**	**490**
SDS1[1]	37	50	29	18
SDS2	18	23	11	14
SDS3	15	17	11	6
SDS4	17	12	20	24
BIS0	**1**	**25**	**0**	**16**
BIS1[1]	37	16	30	18
BIS2	80	90	62	78
BIS3	27	51	6	22
BIS4	35	8	3	20
KPS1	18	74	18	25
KPS2	39	28	15	25
KPS3	38	78	15	29
KPS4[1]	37	16	30	18
KPS5	74	30	30	19

[1] *SDS1, BIS2 and KPS4 are the same suspension.*
